# Mobilization of retrotransposons as a cause of chromosomal diversification and rapid speciation: the case for the Antarctic teleost genus *Trematomus*

**DOI:** 10.1186/s12864-018-4714-x

**Published:** 2018-05-09

**Authors:** J. Auvinet, P. Graça, L. Belkadi, L. Petit, E. Bonnivard, A. Dettaï, W. H Detrich, C. Ozouf-Costaz, D. Higuet

**Affiliations:** 1 Laboratoire Evolution Paris Seine, Sorbonne Université, Univ Antilles, CNRS, Institut de Biologie Paris Seine (IBPS), F-75005 Paris, France; 2 Plateforme d’Imagerie et Cytométrie en flux, Sorbonne Université, CNRS, - Institut de Biologie Paris-Seine (BDPS - IBPS), F-75005 Paris, France; 3 Institut de Systématique, Evolution, Biodiversité (ISYEB), Museum National d’Histoire Naturelle, CNRS, Sorbonne Université, EPHE, 57, rue Cuvier, 75005 Paris, France; 4 Institut Pasteur, Laboratoire Signalisation et Pathogénèse, UMR CNRS 3691, Bâtiment DARRE, 25-28 rue du Dr Roux, 75015 Paris, France; 5 Department of Marine and Environmental Sciences, Marine Science Center, Northeastern University, Nahant, MA 01908 USA

**Keywords:** *Trematomus*, Chomosomal rearrangements, Speciation, Nototheniidae, Retrotransposons, FISH, *DIRS1* insertion hot spots

## Abstract

**Background:**

The importance of transposable elements (TEs) in the genomic remodeling and chromosomal rearrangements that accompany lineage diversification in vertebrates remains the subject of debate. The major impediment to understanding the roles of TEs in genome evolution is the lack of comparative and integrative analyses on complete taxonomic groups. To help overcome this problem, we have focused on the Antarctic teleost genus *Trematomus* (Notothenioidei: Nototheniidae), as they experienced rapid speciation accompanied by dramatic chromosomal diversity. Here we apply a multi-strategy approach to determine the role of large-scale TE mobilization in chromosomal diversification within *Trematomus* species.

**Results:**

Despite the extensive chromosomal rearrangements observed in *Trematomus* species, our measurements revealed strong interspecific genome size conservation. After identifying the *DIRS1*, *Gypsy* and *Copia* retrotransposon superfamilies in genomes of 13 nototheniid species, we evaluated their diversity, abundance (copy numbers) and chromosomal distribution. Four families of *DIRS1*, nine of *Gypsy*, and two of *Copia* were highly conserved in these genomes; *DIRS1* being the most represented within *Trematomus* genomes. Fluorescence in situ hybridization mapping showed preferential accumulation of *DIRS1* in centromeric and pericentromeric regions, both in *Trematomus* and other nototheniid species, but not in outgroups: species of the Sub-Antarctic notothenioid families Bovichtidae and Eleginopsidae, and the non-notothenioid family Percidae.

**Conclusions:**

In contrast to the outgroups, High-Antarctic notothenioid species, including the genus *Trematomus*, were subjected to strong environmental stresses involving repeated bouts of warming above the freezing point of seawater and cooling to sub-zero temperatures on the Antarctic continental shelf during the past 40 millions of years *(My)*. As a consequence of these repetitive environmental changes, including thermal shocks; a breakdown of epigenetic regulation that normally represses TE activity may have led to sequential waves of TE activation within their genomes. The predominance of *DIRS1* in *Trematomus* species, their transposition mechanism, and their strategic location in “hot spots” of insertion on chromosomes are likely to have facilitated nonhomologous recombination, thereby increasing genomic rearrangements. The resulting centric and tandem fusions and fissions would favor the rapid lineage diversification, characteristic of the nototheniid adaptive radiation.

**Electronic supplementary material:**

The online version of this article (10.1186/s12864-018-4714-x) contains supplementary material, which is available to authorized users.

## Background

Chromosomal changes are considered by many as a major driving force behind speciation [1–3]. They are major sources of accumulation of genetic incompatibilities, and their fixation is the first stage toward complete reproductive isolation [4, 5]. Through recombination events [6, 7], transposable element (TE) activity can lead to chromosomal rearrangements [2, 8, 9] that may drive lineage-specific diversification [1, 10], although the relative importance of TE-mediated rearrangements to speciation continues to be debated [11, 12]. Therefore, the characterization of TE content and chromosomal organization among diverging lineages in natural specie ensembles is an essential step toward understanding the role of mobile elements in species diversification.

TEs are major components of eukaryote genomes: they impact genome structure and plasticity [7, 13, 14], generating genetic variability on which different evolutionary forces can act [15]. Depending on their mode of transposition, TEs are divided in two categories: the DNA transposons (class II) move through a “cut-and-paste” mechanism, whereas the retrotransposons (class I) replicate via a “copy-and-paste” process involving an RNA intermediate. Five major orders of class I TEs have been identified [16]: Long INterspersed Elements (LINEs), Short INterspersed Elements (SINEs), Penelope (PLEs), Long Terminal Repeat (LTR) retrotransposons and tYrosine Recombinase (YR) encoding elements (also known as *DIRS*, *Dictyostelium* Intermediate Repeat Sequence). Within each order, TEs have been categorized in different superfamilies. In animal genomes, the *Gypsy*, *Copia* and *BEL/Pao* superfamilies of the LTR order are the most widely distributed and diversified [17–19]. These elements are flanked by two direct LTRs that encompass the promoter and regulatory regions. Between the LTRs two open reading frames (ORFs) are usually found [20]: 1) *gag*, encoding virus-like particles; and 2) *pol*, encoding a reverse transcriptase (RT), an RNase H (RH), and an integrase (INT), necessary for insertion activity. The *pol* region of *DIRS* elements are generally similar to LTR retrotransposons, except for the substitution of a tyrosine recombinase ORF for the integrase ORF of LTRs. The *DIRS* order contains *Ngaro*, *VIPER*, and *DIRS* superfamilies; *the last* is further subdivided into *DIRS1* and *PAT* elements. In contrast to LTR retrotransposons found in a wide continuum of species, *DIRS* elements have a more patchy taxonomic distribution [21]. While *DIRS* are absent in model organisms, they have been detected in teleosts (*Danio rerio* and *Takifugu rubripes*) [22–25]. Moreover, the small number of *DIRS* families in the species genomes [26, 27] stands in strong contrast to the abundant representation of *LTR* families [16, 28–30].

Bursts of TE activity and amplification in the genomes of plants [9], *D. melanogaster* [31], and vertebrates [10, 13, 32] are known to have participated in speciation events [33], for example by promoting genomic destabilization and incompatibilities (postzygotic barriers) in hybrid generations [34]. Furthermore, environmental changes, including thermal stress, can cause the breakdown of the epigenetic control that normally represses TE activity [35]. This may cause massive bursts of transposition during species radiations [3, 32, 36]. The activation and mobility of TEs increase chromosomal diversification and potentially speciation rate in some groups, as shown in *D. melanogaster* [37–39], in maize [40], and in primates [41]. However, integrative analyses of chromosomal rearrangements and their linkage to retrotransposition in complete taxonomic groups have in general been hindered by lack of the requisite molecular, chromosomal and environmental data.

During the last 40 millions of years (*My*), the Antarctic fauna have experienced multiple glacial-interglacial cycles, leading to habitat disturbance by iceberg scouring and habitat fragmentation during glacial maxima [42–44]. The Antarctic teleosts fish family Nototheniidae (antifreeze glycoprotein-(AFGP-) producing “cod icefishes” [45]) rapidly diversified into several species flocks and now constitute the dominant group of Antarctic teleosts [45–47] with well-documented phylogenetic relationships [45, 48–50]. The nototheniid genus *Trematomus* (including *Indonotothenia cyanobrancha* [50]) is an example of a relatively recent (− 9.1 *My* [45]; − 4.3 *My* [51]) and rapid marine adaptive radiation. All are endemic to coastal waters of the Antarctic continental shelf. Although their ancestral diploid karyotype was inferred to have 48 acrocentric chromosomes (the typical karyotype of most modern teleosts), trematomines exhibit the highest chromosomal diversity among the Nototheniidae [52, 53]. Diploid chromosome numbers range between 24 and 58, and chromosomal rearrangements of all types are plentiful [54–56], which is rare among other marine teleosts [57]. Sex chromosome differentiation also occurred via genomic restructuring [4, 52, 53, 55], as at least five *Trematomus* species possess a multiple sex chromosome system of the female X1X1X2X2 / male X1X2Y type. Males have an odd chromosome number due to the tandem fusion between one X1 and one X2 autosome to form the Y element.

The tractable species number of the monophyletic genus *Trematomus*, the history of thermal perturbations of the taxon, its remarkable inter- and intraspecific chromosomal diversity, and its plethora of TEs [58–62] make the group ideal to investigate whether mobilization of TEs in response to environmental change may have played a role in the chromosomal diversification that often accompanies rapid speciation events. In the present multi-strategy study, we focus on *DIRS*- and LTR-order retrotransposons, specifically the diversity, distribution, chromosomal locations, and quantification of the *pol* region of the *DIRS1*, *Gypsy*, and *Copia* elements, which we find to be widely distributed in the genomes of *Trematomus* and other nototheniid species. The results obtained were compared to related temperate and Sub-Antarcic non AFGP-bearing species that have not experienced such environmental changes.

## Results

### Genome sizes estimations

We determined a mean genome size of 1.19 ± 0.12 pg (s.e.m.) for *Trematomus* species, with C-values ranging from 1.09 to 1.26 pg. Our values from the nototheniids *D. mawsoni* (1.02 pg) and *N. coriiceps* (1.36 pg) flanked the trematomine range. Variance between technical triplicates (0.015 pg, s.e.m.) and biological triplicates (0.017 pg, s.e.m.) were correspondingly low (one to seventeen specimens per species, see Additional file 1). In general, our results are in close agreement with those C-values published previously (Table 1).

**Table 1:** C-values (pg) of nototheniid species

Sub-families	*Genus. species* (*abbreviation*)	Chromosomal numbers (2n)	No. examined	C-values (pg) ± SE	Prior published results (ref)
Trematominae	*T. eulepidotus (Teu)*	24	1	1.26	
	*T. pennellii (Tpe)*	32	1	1.15	
	*T. borchgrevinki (Tbo)*	45 – 46 ^a^	8	1.09 ± 0.039	1.28 [116]
	*T. hansoni (Tha)*	45 - 46/46/48 ^a^	12	1.26 ± 0.025	
	*T. bernacchii (Tbe)*	48	17	1.12 ± 0.019	1.20 [73, 117]
	*T. loennbergii (Tlo)*	47 - 48 ^a^	1	1.34	
	*T. lepidorhinus (Tle)*	47 - 48 ^a^	0	ND	
	*T. newnesi (Tne)*	45 - 46 ^a^	2	1.15 ± 0.141	1.01 [60]
	*T. scotti (Tsc)*	48	0	ND	
	*T. nicolaï (Tni)*	57 - 58 ^a^	2	1.17 ± 0.04	
	*I. cyanobrancha (Icy)*	48	0	ND	
Nototheniinae	*N. coriiceps (Nco)*	22	3	1.36 ± 0.118	1.13 [60]
Dissostichinae	*D. mawsoni (Dma)*	48	3	1.02 ± 0.016	0.97 [116]; 1.03 [73, 117];1.20 [60]

^a^Several different karyotypes per species due to sex chromosome differentiation (either an X1X1X2X2 female or an X1X2Y male). The possible combinations from our sampling are underlined

*ND* not determined, no erythrocytes samples available

### Nototheniid retrotransposons: Identification, genomic distribution and characterization

Four families of *DIRS1* (named *YNotoJ*, *V*, *B* and *R*) have been identified based on the 1.25 kb fragments overlapping the RT/RH domains. For the *Gypsy* elements, nine families (named *GyNotoA*, *B*, *D*, *E*, *F*, *H*, *I*, *J*, *RT*) have been identified; five with 1.5 kb fragments than span the RT/RH/INT domains (region 2), the other four with 0.6 kb fragments overlapping the RT/RH or the INT domain (region 1 or 3), as it was not possible to extend the seed by TE-walking. Two families of *Copia* (named *CoNotA* and *CoNotoB*) were detected with sequences of 1 or 1.4 kb overlapping either the RT/RH or the RT/RH/INT domains (Table 2). All identified TE families appeared ubiquitous, in all investigated genomes, with three exceptions: *GyNotoD* and *GyNotoJ*, only detected in one or two genomes; and *CoNotoA* not detected in *Trematomus* genomes (Additional file 2).

**Table 2:** Characteristics of the *DIRS1*, *Gypsy* and *Copia* sequences (TE dataset) detected in nototheniid genomes

TE superfamily	Family	Amplification size (kb)^1^	Region^2^
*DIRS1* (*YNoto*)	*YNotoJ*	1.25	1
	*YNotoV*	1.26	1
	*YNotoB*	1.25	1
	*YNotoR*	1.25	1
*Gypsy* (*GyNoto*)	*GyNotoA*	1.54	2
	*GyNotoB*	1.54	2
	*GyNotoD*	1.55	2
	*GyNotoE*	1.62	2
	*GyNotoF*	0.61	3
	*GyNotoH*	0.61	3
	*GyNotoI*	0.61	3
	*GyNotoJ*	1.22	2
	*GyNotoRT*	0.65	1
*Copia* (*CoNoto*)	*CoNotoA*	0.94	1
	*CoNotoB*	1.38	2

^1^Size of TE consensus from each family found in nototheniid genomes.^2^Either for *DIRS1*, *Gypsy* or for *Copia* elements, we focused on the *pol* region. The TE sequences are then overlapping on 1: RT/RH, 2: RT/RH/INT or 3: INT domains

All TE families sequenced showed a strong intrafamily conservation across nototheniid species. We found a high nucleotide sequence identity within each *DIRS1* family (88.2 to 91.4%) (Table 3a), as well as within each *Gypsy* family (88.3 to 96.6% for the fragments that span the RT/RH portion and 89.7 to 97.8% for the fragments overlapping on INT portion) (Table 3b). *Copia* sequences within a family were similarly conserved (95.9 to 98.5%) (Table 3c).

**Table 3:** Nucleic acid identity matrices of *DIRS1* (a), *Gypsy* (b) and *Copia* (c) families across nototheniid species

a							
**RT/RH**	*YNotoB*	*YNotoR*	*YNotoJ*	*YNotoV*			
*YNotoB*	**88.2**	48.3	49.4	49.6			
*YNotoR*		**91.4**	45.7	50.1			
*YNotoJ*			**88.4**	55.3			
*YNotoV*				**89.8**			
b							
**RT/RH** **INT**	*GyNotoA*	*GyNotoB*	*GyNotoD*	*GyNotoE*	*GyNotoF*	*GyNotoH*	*GyNotoJ*
*GyNotoA*	**96.6**	59.4	60.9	62.8			62.6
	**95.7**						
*GyNotoB*	62.6	**92.2**	68.9	64.5			57.9
		**89.7**					
*GyNotoD*	64.4	70.6	**NA**	64.8			59.4
			**NA**				
*GyNotoE*	65.7	66.3	69.2	**96.6**			60.4
				**96.0**			
*GyNotoF*	65.2	66.2	68.9	74.4	**97.8**		
*GyNotoH*	65.5	61.7	63.3	62.6	63.8	**93.8**	
*GyNotoJ*							**88.3**
c							
**RT/RH ± INT**	*CoNotoA*	*CoNotoB*					
*CoNotoA*	**98.5**						
*CoNotoB*		**95.9**					

Note - Identity percentages are based on the nucleotide sequence alignments of identified nototheniid retrotransposons (see Additional file 9 for taxonomic sampling).TE intrafamily percentage identies across nototheniid species are indicated in **bold**. For *Gypsy* elements, identities calculated from RT/RH are presented above the table diagonal line and those based on INT are shown below. NA: not applicable because only one sequence identified for the family

The maximum divergence between TE families across nototheniid species was 50.3% for *DIRS1*, followed by 37.8% interfamily divergence for *Gypsy*. *CoNotoA* (*GalEa*) and *CoNotoB* (*Hydra*) could not be reliably aligned for comparison.

### Nototheniid retrotransposon positioning among eukaryote TEs

**Fig. 1:**
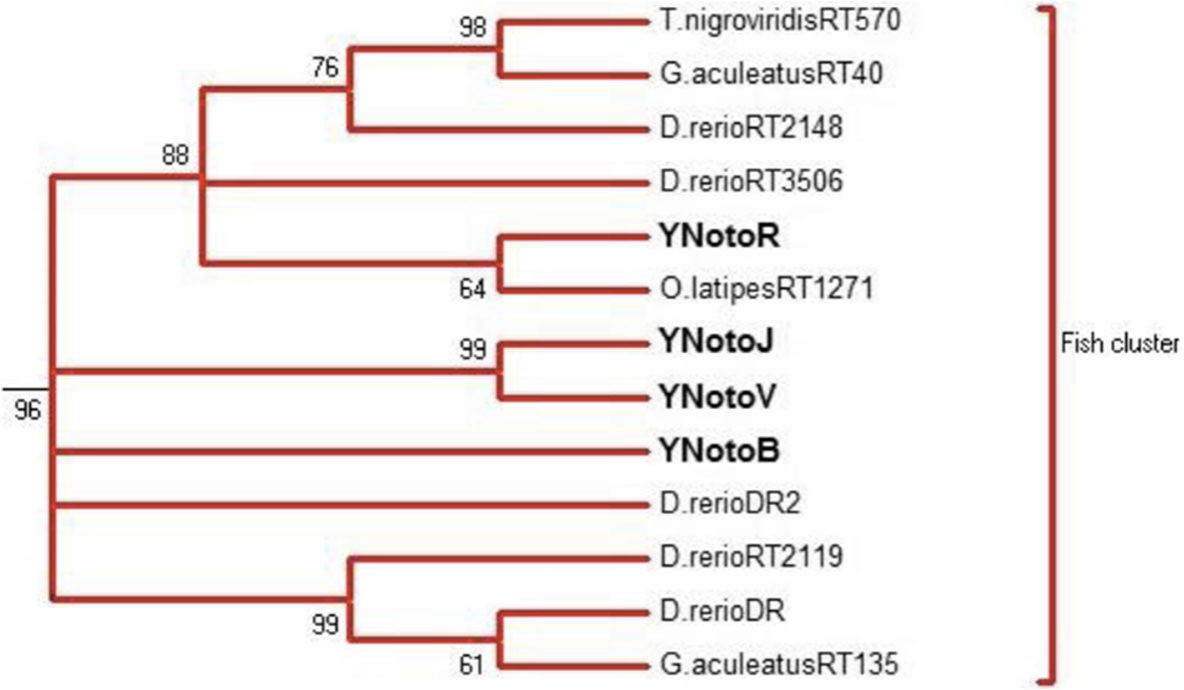
NJ bootstrap consensus tree for *DIRS1* based on the RT/RH amino acid sequences. Only the branch containing the nototheniid *DIRS1* families and the closest related sequences are shown. The four nototheniid *DIRS1* families (**bold font**) group with the other bony fish sequences: *Tetraodon nigroviridis*, *Gasterosteus aculeatus*, *Danio rerio*, *Oryzias latipes*. Distances were calculated with the JTT model and a gamma distribution correction for amino acid. Support for individual clusters was evaluated using non-parametric bootstrapping with 1000 replicates. Only bootstraps over 60 are presented. Nodes with bootstraps < 60% were collapsed. See full tree in Additional file 3

**Fig. 2:**
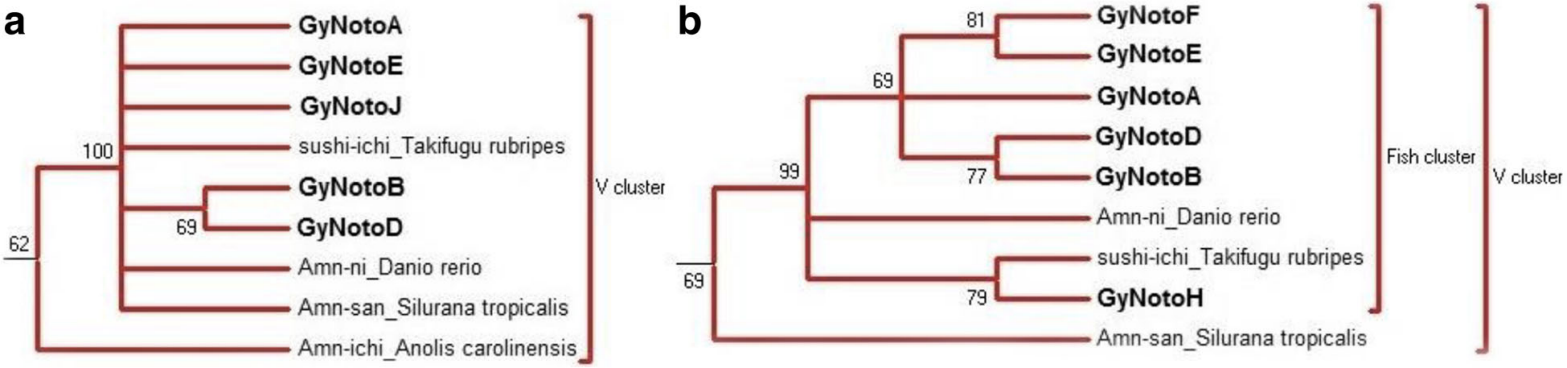
NJ bootstrap consensus tree for *Gypsy* based on the RT/RH (**a**), INT (**b**) regions. Only the branch containing the nototheniid *Gypsy* and the closest related amino acid sequences are presented. Except for *GyNotoI* and *GyNotoRT*, the seven nototheniid *Gypsy* families shown (**bold font**) group with bony fish sequences: *Takifugu rubripes* -sushi-ichi and *Danio rerio* -Amn-ni in addition to other vertebrate *Gypsy* sequences: *Xenopus*/*Silurana tropicalis*. Distances were calculated with the JTT model and a gamma distribution correction for amino acid. Support for individual clusters was evaluated using non-parametric bootstrapping with 1000 replicates. Only bootstraps over 60 are presented. Nodes with bootstraps < 60% were collapsed. See full tree in Additional file 4

**Fig. 3:**
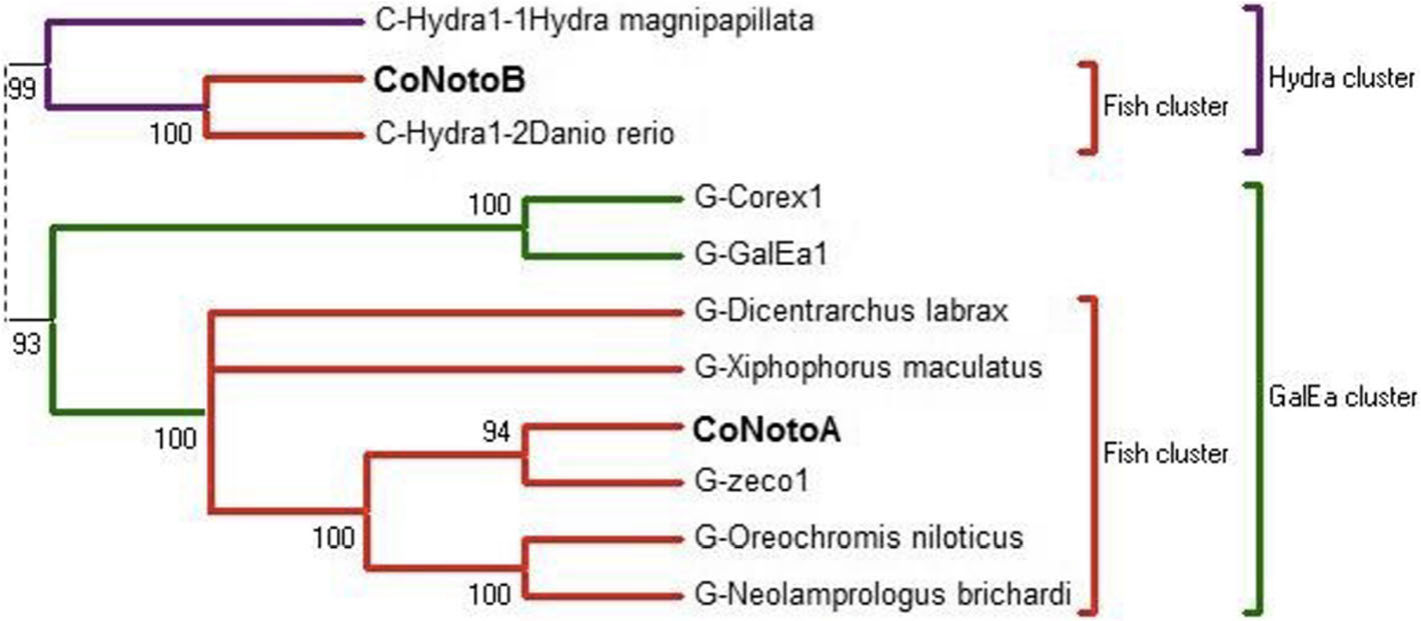
NJ bootstrap consensus tree for *Copia* based on the RT/RH amino acid sequences. Only the branch containing the nototheniid *Copia* families and the closest related sequences are shown. The two nototheniid *Copia* families (**bold font**) identified in nototheniid genomes group with bony fish sequences: *Dicentrarchus labrax*, *Xiphophorus maculatus*, *Danio rerio*, *Oreochromis niloticus* and *Neolamprologus brichardi*. Distances were calculated with the JTT model and the gamma distribution correction for amino acid. Support for individual clusters was evaluated using non-parametric bootstrapping with 1000 replicates. Only bootstraps over 60 are presented. Nodes with bootstraps < 60% were collapsed. See full tree in Additional file 5

We ran phylogenetic analyses for *DIRS1*, *Gypsy* and *Copia* TEs in order to position our TE family among TE families described in eukaryote genomes. The same topologies were observed whether Distance or Maximum Likelihood reconstruction methods were used. The large majority of our sequences cluster together with sequences previously described in bony fish or vertebrate groups (Figs. 1, 2, and 3, Additional files 3, 4, and 5 for full trees). *DIRS1* nototheniid consensus sequences cluster with other bony fish *DIRS1* sequences from *Tetraodon nigroviridis*, *Gasterosteus aculeatus*, *Danio rerio*, and *Oryzias latipes* (0.96 support). Except for *GyNotoI* and *GyNotoRT*, *Gypsy* nototheniid consensus sequences cluster with other vertebrate and bony fish *Gypsy* sequences from *Tetraodon rubripes* and *Danio rerio* (0.62 and 0.69 support, respectively). *Copia* nototheniid consensus sequences cluster with other bony fish - *GalEa* sequences for *CoNotoA* or *Hydra* sequences for *CoNotoB* - from *Danio rerio*, *Dicentrarchus labrax* and *Oreochromis niloticus* (support = 1) (Figs. 1, 2a, b and 3).

All *DIRS1* consensus were positioned among the described “FISH cluster” (“*DrDIRS1*” group) [23, 24, 27]. *YNotoJ* and *YNotoV* seemed to be more closely related between each other than with YNotoR and *YNotoB* (Fig. 1). In the same way, *GyNotoB* and *GyNotoD* were closely related but no nototheniid *Gypsy* clade was detected: all *GyNoto* consensus sequences (except *GyNotoI* and *RT*) were grouped with other *Gypsy* sequences from *T. rubripes* and from *D. rerio* belonging to the described “V cluster” [63] (Fig. 2). *GyNotoI* and *GyNotoRT* even fell outside the bony fish TE clade (Additional file 4) and were much more difficult to align than the other family consensus sequences. Finally, the two *CoNoto* consensus sequences were placed within two distinct *Copia* bony fish sub-clades, both closely related to *Copia* sequences found in the zebrafish *D. rerio*: *Hydra*1-2 for *CoNotoB* and *GalEa*-Zeco1 for *CoNotoA* (Fig. 3).

### Chromosomal location of nototheniid retrotransposon families

**Fig. 4:**
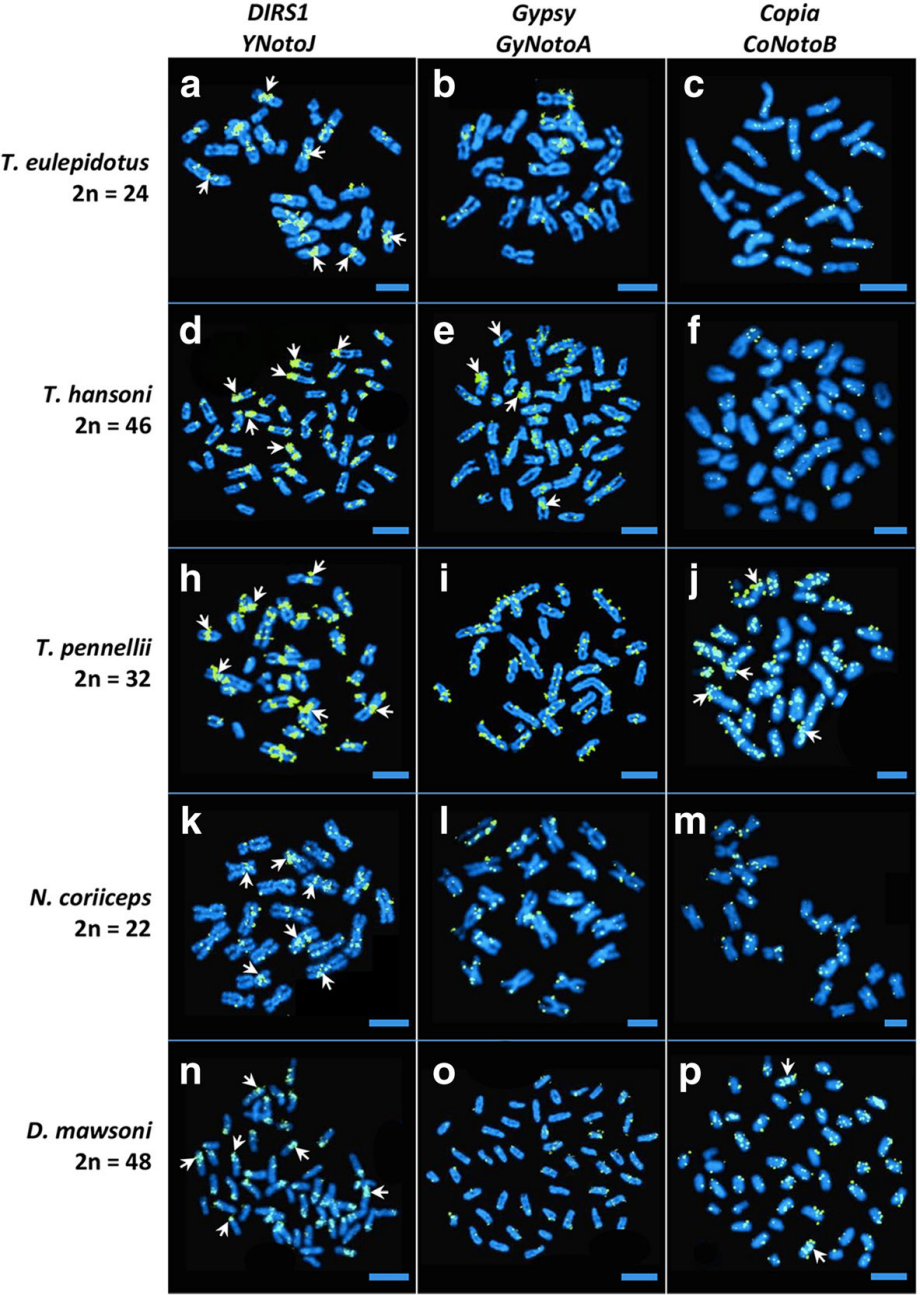
Mapping of TEs on the chromosomes of five nototheniid species by FISH. Each probe was labeled with biotin and bound probe was detected with incubation with Avidin-FITC (fluorescein, greenish spots). (Probe characteristics are indicated in Additional file 6). Chromosomal DNA was counterstained with DAPI. One family of each retrotransposon superfamily is represented in this figure: *YNotoJ* for *DIRS1*, *GyNotoA* for *Gypsy* and *CoNotoB* for *Copia* elements. (see Additional files 7 and 8 for FISH mapping with the second family of *DIRS1* (*YnotoR*) and *Gypsy* (*GyNotoE*)). Examples of TE distribution patterns for type 1: **d**, **h**; type 2: **c**, **i**; type 1 + 2: **e**, **j**, **p**. White arrows point examples of TE accumulations. Scale bars: 10 μm

Fluorescent In Situ hybridization (FISH) is a powerful technology for imaging the distribution of repetitive gene families on the condensed chromosomes of metaphase spreads. To assess the potential role of nototheniid TEs in mediating chromosomal rearrangements in *Trematomus* and other High Antarctic species, we used FISH to map the locations of two families of *DIRS1*, two families of *Gypsy*, and one family of *Copia* (*Hydra*) on chromosome preparations from five nototheniid species that represent the diversity of nototheniid karyotypes (2n = 22 metacentrics to 2n = 48 acrocentrics): *T. eulepidotus, T. hansoni*, *T. pennellii*, *N. coriiceps*, and *D. mawsoni*. For comparison, we chose chromosome preparations from three cool temperate/temperate outgroup species: the Sub-Antarctic notothenioids *B. diancanthus* and *E. maclovinus*, and the Eurasian perch *P. fluviatilis*, all of which feature 48 acrocentric chromosomes. The TE families were chosen for their cloned sequence sizes (> 1 kb) and their ubiquity in the genomes of nototheniid species (Additional files 2 and 6). Figure 4 shows the two major types of TE distribution patterns: (1) dense accumulation (“hot spots”) mainly in centromeric and/or pericentromeric regions and sometimes in intercalary or telomeric positions; and (2) scattered, punctuate staining along chromosome arms. Combinations of these two patterns were also evident. The distributions (1) and/or (2) clearly depended upon the TE super-family, but within a super-family, different TE families produced comparable results (e.g., the nearly identical patterns observed between *YNotoJ* and *YNotoR* of the *DIRS1* superfamily as shown with the double FISH-mapping (Additional files 7 and 8), and between *GyNotoA* and *GyNotoE* of the *Gypsy* superfamily) (Fig. 4 and Additional file 7). For a given TE family, there were small variations of signal intensity or distribution along chromosomes between species, but generally, the same location pattern was observed.

**Fig. 5:**
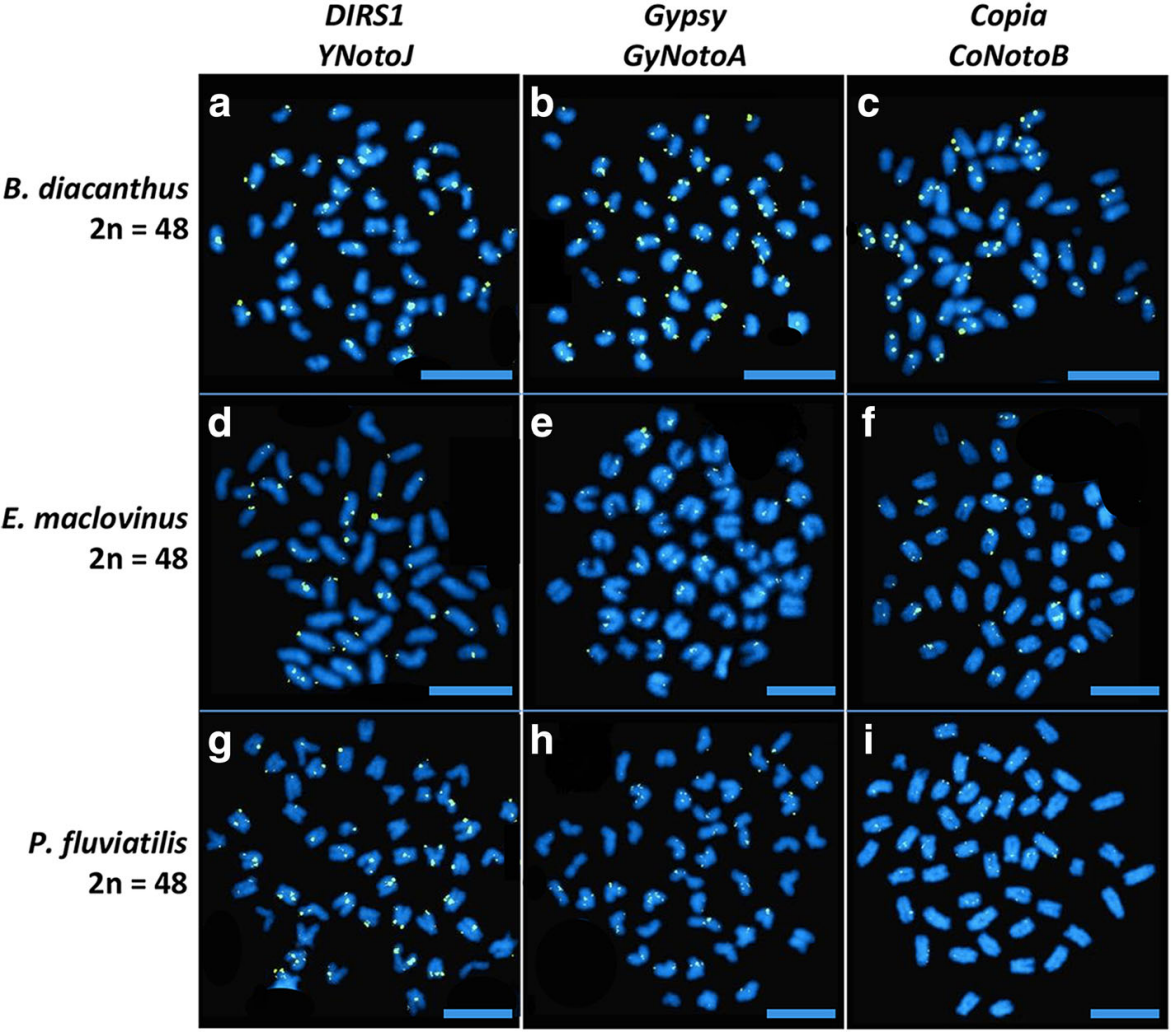
Mapping of TEs on the chromosomes of the three outgroups by FISH. Each probe was labeled with biotin and bound probe was detected with incubation with Avidin-FITC (fluorescein, greenish spots). (Probe characteristics are indicated in Additional file 6). Chromosomal DNA was counterstained with DAPI. One family of each retrotransposon superfamily is represented in this figure: *YNotoJ* for *DIRS1*, *GyNotoA* for *Gypsy* and *CoNotoB* for *Copia* elements. Scale bars: 10 μm

The two investigated families of *Gypsy* and *Copia Hydra* elements were mostly dispersed throughout nototheniid chromosome arms (type 2), forming multiple spots scattered everywhere on nototheniid chromosome arms (*T. pennellii*, *T. eulepidotus*, *N. coriiceps* and *D. mawsoni*) as well as in centromeres and telomeres of acrocentric (*T. hansoni*, *T. pennellii* and *D. mawsoni*) and metacentric (*T. eulepidotus*, *T. pennellii* and *N. coriiceps*) chromosomes. Signals occasionally accumulated on metacentric chromosomes (giving a type 1 + 2 pattern) (Fig. 4e, j, p). In the nototheniid species, the two FISH-investigated families of *DIRS1* elements appeared to accumulate (white arrows in Fig. 4 and Additional file 7) in centromeric regions (type 1), mostly in short acrocentric pairs (Fig. 4d, n), in some telomeres (Fig. 4d, h) and were also aggregated in pericentromeric areas (type 1), especially in karyotypes with metacentric chromosomes (Fig. 4h, k). The *DIRS1* element pattern (type 1) was very distinct from the *Gypsy* and *Copia* patterns, with clear hot spots of insertion in all nototheniid species studied. In contrast, these *DIRS1* hot spots were not detected in the three outgroups *B. diancantus*, *E. maclovinus* and *P. fluviatilis*, where FISH signals were mostly scattered on chromosome arms (type 2), with some of them located on telomeres, but never aggregated. Even if the *Gypsy* and *Copia* FISH signals seemed less abundant, they were still scattered on chromosome arms (type 2) like for the five High Antarctic species (Fig. 5).

### Retrostransposon quantification

The same five TE families (*YNotoJ*, *YNotoR*, *GyNotoA*, *GyNotoE*, *CoNotoB*) were quantified in the same five nototheniid species (*T. pennellii*, *T. hansoni*, *T. eulepidotus*, *N. coriiceps*, *D. mawsoni*) using qPCR. Our quantification results indicated that *Gypsy* and *Copia* elements were present in 7-90 copies per family studied, while *DIRS1* elements were generally represented by 100-150 copies per family in the five genomes (Table 4). For the *DIRS1* retrotransposons, *YNotoJ* and *YNotoV* appeared the most represented families in *T. hansoni*, *T. eulepidotus*, *N. coriiceps* and *D. mawsoni* genomes. In *T. pennellii*, we estimated 370 *YNotoJ* but a roughly equal number for *YNotoV* (110) and *YNotoRB* (125). The *GyNotoA* (between 10 and 90 copies) were more represented than *GyNotoE* (< 10 copies, apart from for *T. hansoni*, with an estimate of 30 copies detected) except in *N. coriiceps* (8 *GyNotoA* vs 20 *GyNotoE*). *T. pennellii* carried the highest number of *DIRS1* retrotransposons when counting all families together (total of 605 estimated copies) and *T. hansoni*, the highest number of *Gypsy* elements (total of 120 estimated copies). Both of those genomes also contained the highest copy number for *Copia Hydra* elements (45 estimated copies) whereas we estimated a low number of copies, 10 to 15 for the other three species *T. eulepidotus*, *N. coriiceps* and *D. mawsoni*.

**Table 4:** TE copy numbers estimated in the nototheeniid genomes

TEs	*DIRS1*	*DIRS1*	*DIRS1*	*Gypsy*	*Gypsy*	*Copia*
Species	*YNotoJ*	*YNotoV*	*YNotoRB*	*GyNotoA*	*GyNotoE*	*CoNotoB*
*T. pennellii*	370	110	125	50	8	45
*T. hansoni*	275	200	30	90	30	45
*T. eulepidotus*	250	80	55	45	7	15
*N. coriiceps*	80	135	15	8	20	10
*D. mawsoni*	120	340	10	20	7	10

## Discussion

### TE diversity

Our investigation revealed a high diversity of retrotransposons within nototheniid genomes, with four families of *DIRS1*, nine of *Gypsy* and two of *Copia* identified. This result corroborates previous studies on the large number and repartition of TEs in teleost species [19, 64, 65]. Within nototheniid species, Detrich et al. [60] have described TEs in *Notothenia coriiceps* and *Chaenocephalus aceratus* from partial genome sequencing data, finding respectively 13.4 and 14.5% of their genomes composed of a wide TE diversity. Retrotransposons seem more represented than DNA transposons.

The present study shows retrotransposon sequence conservation between species, with levels of interspecific nucleotide identity similar to those observed between *Trematomus* species for *Rex1* and *Rex3* non-LTR retrotransposons [59], or for nuclear coding genes like Pkd1 (98.4%), RPS71 (98.2%) and Rhodopsin (98.9%) [45, 50]. This result suggests that these TEs are subject to strong selective pressure to maintain their activity [65, 66]. They also show that transposition events may have occurred recently in those genomes. These observations might be subject to some biases due to the method employed to detect the conserved domains on *pol* region and thus, preferentially potentially active, or recently active TEs.

The presence of twelve of the fifteen identified TE families in all the studied genomes points to a divergence of the TE families prior to the divergence of the nototheniid species [58]. Our sampling includes species representing the oldest divergence in the family, estimated between − 22.4 and − 13.4 My depending on the study [45, 51]. Their mobilization in the genomes however could have occurred more recently, perhaps during nototheniid radiation events.

Although our nototheniid TE consensus sequences belong systematically to the Vertebrate or the fish clade, they are not grouped together like in other studies [59]. All species studied were found to contain at least one copy of each identified TE family, except two families of *Gyspy* (*GyNotoD*, and *GyNotoJ*) and one of *Copia GalEa* (*CoNotoA*). This last family was not recovered in the genus *Trematomus*, but it was successfully amplified in *N. coriiceps*, *D. mawsoni* and other Nototheniidae (Additional file 2). We extended the search for *GalEa* to other trematomine species closely related to *Trematomus*: genera *Indonotothenia*, *Lepidonotothen* and *Patagonotothen* without being able to successfully amplify them by PCR. We are aware that a lack of PCR amplification of a given DNA fragment does not mean it is absent from the genome examined. However, given the strong interspecific conservation of *GalEa* family in nototheniids and the tendency of *GalEa* elements to be secondary lost in entire clades (for example within Fungal and Crustacean genomes [67, 68]), this would indicate the presence of *GalEa* retrotransposons in the last common ancestor of all Nototheniidae was followed by a secondary loss in Trematominae. Such result supports recent phylogenetic studies grouping together the genera *Cryothenia*, *Indonotothenia*, *Pagothenia*, *Trematomus, Lepidonotothen* and *Patagonothen* in the Trematominae sub-family [45, 48–51, 69, 70].

### Extensive chromosomal rearrangements and conserved genome size within the Trematomus

*Trematomus* C-values for the different studied species are very close to each other (1.0 to 1.2 pg). They fall also in the same range as the values obtained or published for other nototheniid sub-family representatives: Notothenia (*N. coriiceps*, 1.36 pg [60]), Dissostichus (*D. mawsoni*, 1.02 pg [60]), Lepidonotothen (*Lepidonotothen nudifrons*, 1.12 pg [60]), and Gobionotothen (*Gobionotothen gibberifrons*, 0.98 pg [71]).While there is no available genome size data for the sister groups of Nototheniidae (Bovichthidae, Eleginopsidae or Pseudaphritidae), or for the most recently proposed sister group of Notothenioidei, Percophidae [72], the genome sizes of other families phylogenetically close to the suborder Notothenioidei like Percidae (*P. flavescens*, 0.92 pg, *P. fluviatilis*, 0.90 pg, *Percina caprodes*, 1.06 pg, *Sander lucioperca*, 1.14 pg, *Sander vitreus*, 1.06 pg) [60, 73], or Serranidae (1.09 pg mean genome size, 0.17 s.d) [74] also range between 0.90 and 1.23 pg. This suggests that a C value of 1-1.20 pg could be the ancestral state, conserved throughout the *Trematomus* radiation. Therefore, karyotype diversity within the genus *Trematomus* occurred without much variation in the amount of DNA per genome. Rearrangements involving changes in *Trematomus* chromosome numbers and formulae are structural modifications like tandem or Robertsonian centric fusions (2n = 24 in *T. eulepidotus*, 2n = 32 in *T. pennellii* and 2n = 45–46 in *T. hansoni*, *T. borchgrevinki* and *T. newnesi*) or fissions (2n = 57–58 in *T. nicolaï*) and maybe inversions. However, inversions could not be detected using FISH or classical chromosome banding tools [54, 55].

Genome size stability is not observed in all nototheniid species. We also measured the C-values of some nototheniid species from the sub-families Channichtyinae, Gynmodraconinae and Bathydraconinae, phylogenetically closely related to each other, but further from the Trematominae. Although few species have been measured yet, they display higher C-values: 1.72 pg for *Chionodraco hamatus*, 1.54 pg for *Gymnodraco acuticeps*, and 1.34 pg for *Parachaenichtys charcoti*. This noticeable genome size augmentation could be explained by massive transpositions, segmental or genome duplication that could have happened during diversification of these groups [2, 18, 71].

### Did TE mobilization favor chromosomal diversification during the Trematomus radiation?

To evaluate the representation of some *DIRS1*, *Gypsy*, and *Copia* families in nototheniid genomes in the absence of a well-assembled genome available to run exhaustive computational analyses, we tried to estimate their copy number using qPCR approaches relative to two single copy gene standards. Despite the repeated nature of the TE sequences, this approach has already been used to estimate TE copy number on genomic DNA [62, 75, 76]. The same TE families quantified by qPCR were also mapped on nototheniid chromosomes using FISH. Even if FISH is not precise enough to estimate accurate TE copy numbers, we could estimate their relative number by the intensity of signals (Figs. 4, 5, Additional files 7, 8). There is a consistency between the two approaches that is generalizable to the five nototheniids studied (numerous hot spots and high copy number, few interspersed spots and low number of copies) (Table 4, Fig. 5, Additional file 7). In at least a few cases, there is probably a tendency for the qPCR to underestimate the real copy number, as based on their intensity most FISH signals correspond to multiple copies. This is particularly obvious for the *Hydra* (*CoNotoB*) probe as there are multiple spots (certainly more than the estimated 10 copies) present in all chromosomes of *N. coriiceps* and *D. mawsoni* (Fig. 4, Table 4). The regions amplified by qPCR are only a portion of the TE sequence used as probe for FISH; the signals are therefore not totally comparable. Whole or partial genome sequencing would be needed to get more precise estimations of relative weights of TE families in those genomes and their respective true copy numbers.

The copy number estimated for *DIRS1* elements is higher than for *Gypsy* and *Copia* retrotransposons in all five genomes quantified. It cannot, however, be excluded that the quantification of *Gypsy* elements is more biased by unequal amplification because of their higher family diversity (we quantified two of the four identified *DIRS1* families vs two of the nine identified *Gypsy* families). This tendency of *DIRS1* prevalence is even more pronounced for the three *Trematomus* species studied, *T. eulepidotus*, *T. hansoni* and *T. pennellii*. The predominance of *DIRS1* copies over *Gypsy* and *Copia* copies is contrary to the strong prevalence of *Gypsy* previously reported in the *Takifugu* genome (10 putative copies of *DIRS*, 35 copies of BEL/Pao, 50 copies of *Copia* and 2500 copies of *Gypsy* identified) [64], in the zebrafish genome (46,806 copies of *DIRS*, 4585 copies of BEL/Pao, 10,293 copies of *Copia* and 56,138 copies of *Gypsy* identified) [25], and in two nototheniid genomes based on partial sequencing of a BAC library (for *N. coriiceps*, 17 copies of *DIRS*, 1 copy of *Copia* and 49 copies of *Gypsy* identified, for *C. aceratus*, 46 copies of *DIRS*, 25 copies of *Copia* and 156 copies of *Gypsy* identified, no BEL/Pao identified in both cases) [60, 71]. The *DIRS1* prevalence in *Trematomus* and other High Antarctic species investigated is consistent with the observed FISH signals (Fig. 4). As it has been proposed in several studies, strong multiplication of retrotransposons could have happened during chromosomal diversification accompanying speciation events [1, 13, 18, 58, 59, 77–80]. The bursts of retrotransposons could therefore have occurred during the radiation of *Trematomus* and other AFGP-bearing nototheniid relatives (Chaniichthyinae, Artedidraconinae).

The possible implication of massive TE mobilization in the observed *Trematomus* and nototheniid species chromosomal rearrangements is dependent on their location on the chromosomes. We noticed a major difference of *DIRS1* insertion patterns compared to those of *Gypsy* or *Copia* elements. *Gypsy* and *Copia* have numerous and scattered interstitial hybridization spots on chromosomes with no clear regions of accumulation, a pattern comparable to those previously reported for *Tc1*-like DNA transposons [58] and for *Rex*-like non-LTR retrotransposons [59] with mappings on nototheniids chromosomes (including *T. bernacchii*, *T. hansoni*, *T. newnesi* and *T. pennellii*). In striking contrast, *DIRS1* elements accumulate in hot spots of insertion mostly located in centromeric and pericentromeric regions (Fig. 4) for the several nototheniid radiations studied, including in *Chionodraco hamatus* (sub-family Channichthyinae). In the biggest chromosome pair of *T. pennelli*, which certainly arose from two fusions (C. Ozouf-Costaz, unpublished data), there is an intercalary band of *DIRS1* in the long arm in addition to the pericentromeric hot spot. This pattern of insertion is reminiscent of the one highlighted by Ozouf-Costaz et al. with *Rex3* retrotransposon on the Y chromosome (originating from a tandem fusion between one X1 and one X2 autosome) of *C. hamatus* [59]. The intercalary band observed in the long arm of this Y chromosome suggest *Rex3* involvement in the tandem fusion (telomere-centromere) between the two X1 and X2 autosomes this chromosome originates from [53, 56].

*DIRS1* are not structured like LTR retrotransposons and this specificity may be linked with it particular origin. Discovered in the slim mold *Dictyostelium discoideum* [23], *DIRS1* might have arisen from the combination of a crypton-like transposon using tyrosine recombinase (YR) to cut and rejoin the recombining DNA molecules and the RT/RH part of a pre-existing *Gypsy* LTR retrotransposon [22]. Various molecular mechanisms for *DIRS1* insertion in targeted sites (by integration, recombination) have been proposed but remain to be experimentally demonstrated [23, 24, 26]. A specific insertion mode was deduced [81] from this singular molecular structure: via a double-stranded circular pre-integrative DNA intermediate, catalyzed by the YR. This explains the absence of Target Site Duplication (TSD) following transposition [78, 82]. This is reminiscent of bacterial processes and could lead to ectopic recombination [2, 7] and thus, to rearrangements. Hot spots of insertion for DNA transposons (*Tc1*-like) and retrotransposons (*Rex*-like) have already been described a number of times in the literature for other teleost fish groups [58, 65, 80, 83, 84]. *DIRS1* clustering in hot spots of integration is probably not random. Many *DIRS1* elements appear to have preferential target site selection and insert into their pre-existing copies in *Danio rerio* [85, 86]. This process, also called “homing”, is usually reported for class II TEs, such as the *Drosophila* P element [87] and, to our knowledge, has been rarely reported for retrotransposons. Like the other TEs localized in teleost fish chromosomes [58, 65, 80, 83, 84], *DIRS1* inserts in « sheltered » heterochromatic regions [31, 80, 84, 88, 89]. Such locations in regions less affected by selection and with lower recombination rates might protect the TEs [90]. It might also has been selected as a way to protect and structure genomes, ensuring a kind of self-control of their mobilization [1, 20, 65, 83].

A breakdown of epigenetic control mechanisms [2, 3, 36] could have favored *DIRS1*, *Gypsy*, and *Copia* activation, both in *Trematomus* species and other AFGP-bearing nototheniid relatives. According to recent time-calibrated molecular phylogeny analyses [45, 51], the *Trematomus* species diversification occurred in the middle – late Miocene (− 17 to − 5 My), corresponding to a cooling of shallow Antarctic shelf waters, followed by rapid switches between glacial and interglacial conditions [42–44, 91]. This could have led to transpositional waves through genomes [11, 21, 62] possibly linked to species diversification [2, 3]. On the contrary, *B. diacanthus*, *E. maclovinus*, *P. fluviatilis* (non AFGP-bearing species chosen as outgroups) were not exposed to series of environmental changes with cooling at sub-zero temperatures [42, 43], and do not reveal any regions of TE accumulation on their chromosomes for the studied TEs (Fig. 5). TE hot spots were not detected in Bovichtidae (including one species from the subantarctic Southern Ocean), or in the two temperate species from the families Eleginopsidae and Percidae (including a few species that originated in South America, Southern Australia/Tasmania and New-Zealand) [70]. Their karyotypes (2n = 48, all acrocentric chromosomes [92–94]) are very stable across species [92, 95], suggesting few structural chromosomal rearrangements.

## Conclusions

Transposable elements constitute key points to explore and understand genome evolution, especially in the recent divergence of Antarctic *Trematomus* species. While our TE exploration is wider than in previous studies, we cannot yet conclude the lack of detection of a given TE or family in a genome means they are absent. Targeted amplicon shotgun sequencing and untargeted genome sequencing are needed for more exhaustive exploration of TE populations and diversity.

No bursts linked with TE multiplication and accumulation on chromosomes were detected on the three temperate and subantarctic outgroups without AFGPs. On the contrary, exposure to strong environmental changes with cooling at sub-zero temperatures and series of glaciation and deglaciation cycles could have led to massive mobilization of retrotransposons (epigenetic deregulations), observed within *Trematomus* and other nototheniid genomes. A predominance of *DIRS1* (probably several hundred of copies) in hot spots of insertion suggests they could have facilitated repeated and localized DNA double strand breaks on centromeric and pericentromeric regions. Then, the cellular repair mechanisms, close to recombination processes, might have favored tandem or centric fusions and boosted other genomic changes [35, 96]. Chromosomal rearrangements reinforce reproductive isolation between distinct populations and drive vertebrate lineage diversification. They may have accompanied the *Trematomus* radiation.

Like the repeated independent emergences of the male Y-chromosome from centric fusions or tandem translocations, the chromosomal rearrangements observed in *Trematomus* species, and more generally in Nototheniidae, probably occurred independently as the result of convergent events. However, precise reconstruction of the evolutionary history of *Trematomus* chromosomal diversification requires identifying the inter-specific homologies between chromosomes. In this group, these homologies are generally impossible to establish due to the resemblance between chromosomes when using karyotype approaches based on chromosome morphology or size, and on DAPI counterstaining. Since it is impossible to recognize the chromosomes by classical techniques like banding, the development of tools like chromosome painting or BAC-FISH in these species would help to ascertain these inter-specific homologies [4], permitting a more precise history of the genome reorganizations (evolutionary scenario) in this group, and their possible relation with the speciation events, to be established.

## Methods

### Fish specimens

Specimens of thirteen High Antarctic nototheniids (*Trematomus eulepidotus, T. pennelli, T. borchgrevinki, T. hansoni, T. bernacchii, T. loennbergi, T. lepidorhinus, T. newnesi, T. scotti, T. nicolaï, I. cyanobrancha, Notothenia coriiceps, and Dissostichus mawsoni*) and of two Sub-Antarctic notothenioid outgroups *(Bovichtus diacanthus, Eleginops maclovinus*) were collected during groundfish survey programs sponsored by the IPEV (Institut Polaire français Paul-Emile Victor) and the NSF (U.S. National Science Foundation): the ICEFISH 2004 Cruise (International Collaborative Expedition to collect and study Fish Indigenous to Sub-antarctic Habitats, Atlantic sector of the Southern Ocean), ICOTA (Icthyologie côtière en Terre Adélie, 1996-2008, Adelie Land), CEAMARC (Collaborative East Antarctic Marine Census for the Census of Antarctic Marine Life; 2007-2008, Eastern Antarctica, north of Adélie Land and George V Land), POKER (POissons de KERguelen, 2006, 2010 and 2013, shelf of Kerguelen-Heard islands) and REVOLTA (Ressources Ecologiques et Valorisation par un Observatoire à Long terme en Terre Adélie, 2010-2014, Adelie Land). Specimens of *Perca fluviatilis* were obtained in 2010 in Eaucourt (Somme, FRANCE) and are part of the MNHN collection. Tissue samples, chromosomal preparations and blood cells are referenced in Additional file 9. We used the nomenclature and classification of the SCAR atlas [70].

### Sample collection and preparation

#### Blood samples for flow cytometry

Fish were anesthetized with MS 222 before sampling blood (minimum 0.5 ml) by caudal venipuncture using heparinized syringes. Whole blood was diluted 1:10 with PBS, 50 μl aliquots of each blood sample were distributed dropwise to Eppendorf tubes containing 1 ml of 70% ice cold ethanol, and the tubes were stored at − 20 °C.

#### Tissues for DNA analyses

Muscle samples or fin clips for DNA analyses were stored in 85% ethanol at − 20 °C. DNA was prepared following the protocol of Winnepenninckx et al. [97].

#### Chromosome preparations

Mitotic chromosome preparations were obtained from primary cell cultures of pronephric kidney or spleen according to Rey et al. [98]. Briefly, a suspension of cephalic kidney and/or spleen cells was prepared and cultured in L-15 Leibovitz culture medium without bicarbonate and supplemented with L-glutamine, fetal calf serum, lectins (concanavalin A and pokeweed mitogen) and antibiotics for periods up to one week at 0° to 2 °C. Colchicine was added 6 h prior to harvesting cells. This was followed by a hypotonic treatment (1 h at 2 °C) and conventional steps of fixation. Fixed cell suspensions were preserved as aliquots of 15 ml at − 20 °C. Prior to use, the suspensions were thawed and centrifuged at 1500 rpm (Eppendorf 5430 microcentrifuge) for 10 min at 4 °C. After decanting the supernatant, the cell pellet was resuspended in 0.8-1 ml of fresh fixative, and cells were spread onto Superfrost slides (pre-cleaned with absolute ethanol containing 1% of 1 N HCl). Slides were stored at − 20 °C until the FISH step.

Additional file 9 summarizes the blood samples, tissue samples, and chromosomal preparations used in this study.

### Retrotransposon amplification and fragment assembly

#### Amplification of retrotransposons using degenerate primers

Retrostransposons were amplified from the genomes of all *Trematomus* species, with the exception of *T. tokarevi*, and from two nototheniid sister species, *N. coriiceps* and *D. mawsoni* (Additional file 9). Rather than pursue full-length elements (5 to 10 kb), we focused on their *pol* regions, as they include conserved domains and have a fundamental function in transposition. We amplified the retrotransposon *pol* fragments by PCR using degenerate oligonucleotide primer pairs designed for conserved protein motifs of the Reverse Transcriptase/RNAseH (RT/RH) domains (Additional file 10). For *Gypsy* retrotransposons, the GD1b/GD2b primer pair overlaps the PFLG/DASXXGW motifs. CD1 had been designed to amplify *Copia* elements when used with CD2, as previously employed for the galatheid squat lobsters [99], but in nototheniid fishes, we found that it amplified the *Gypsy* Integrase gene, serving as both forward and reverse primer. For *Copia* elements, two primer pairs were used (CD3/CD4, CD5/CD6), which correspond to the DYCYR/DNQG and VDP/QLAD motifs, respectively. For *DIRS1* retrotransposons, we used the DD10/DD11 pair, which encode the DlkdAY/YafPPf motifs.

PCR was performed using 50 ng of genomic DNA, 2.5 U of Taq DNA polymerase (Promega) and 50 pmol of each degenerate primer in a final volume of 25 μL for 35 cycles (94 °C for 45 s, 50.2 °C for 1 min and 72 °C for 1 min). PCR products were visualized on 1% agarose gels. Fragments of the expected molecular weights were excised, purified with the Nucleospin Extraction kit (Macherey_Nagel), and cloned into the pGEM-T vector according to the supplier’s recommendations (Promega, Madison, WI, USA). Cloned fragments were sequenced in both directions (http://www.gatc-biotech.com).

#### TE walking

Optimal DNA fragment sizes for classification of retrotransposons and for use as FISH probes, in our experience, range between 1.0 and 1.5 kb. The initial *Gypsy* amplicons (0.7 kb), in particular, did not meet this standard (Additional file 10). Therefore, we used “TE Walking” [21], anchored by specific primers designed from the original sequences and the GD4 degenerate primer (overlaps the YLDD motif), to extend the *Gypsy* fragments (Additional files 10 and 11). *Copia* and *DIRS1* elements were extended in similar fashion. Each new sequence was manually validated as an extension of the original fragment using a minimum overlap of 50 bp between the two sequences and a minimum DNA identity of 95%. Final retrotransposon sequences were assembled from their fragments using the Cap contig assembly program included in BioEdit v7.2.5 [100]. We then designed specific primer pairs that we used to amplify the desired *pol* fragments (Additional file 11). We obtained DNA cloned fragments from 1.0 to 1.5 kb size, depending on the TE element. Consensus sequences were deposited in BankIt (BankIt2016770: MF142597-MF142757). These larger DNA fragments were used for classification of retrotransposons and as FISH probes.

### Retrotransposon classification, clustering, and phylogenetic analyses

#### Classification and clustering of retrotransposon families

Nototheniid TEs were assigned to a specific retrotransposon superfamily using BLASTX analyses [101] of a custom in-house TE database (2.2.28 + blastpackage), the NCBI protein database, and the *N. coriiceps* genome [102]. *DIRS1*, *Gypsy*, or *Copia* sequences were clustered using BLASTClust toolkit v2.2.26 [103]. A cluster of sequences was considered a separate family if its highest intra-group divergence was lower than the divergence between all putative families, without overlap of the two distributions [21].

The criteria for inclusion of a fragment in a cluster (i.e., family) were ≥ 30% sequence coverage and ≥ 80% sequence identity. We adopted the following nomenclature: *YNotoJ*, *V*, *R*, and *B* are the four identified families of *DIRS1*; *GyNotoA*, *B*, *D*, *E*, *F*, *H*, *I*, *J*, and *RT*, the nine identified families of *Gypsy*; *CoNotoA* and *CoNotoB*, the two identified families of *Copia*. Nucleic acid sequence identity matrices were calculated after alignment of sequences by MAFFT v7 [104] and gap removal using BioEdit v7.2.5.

#### Phylogenetic analysis of nototheniid retrotransposons

The sequences of the nototheniid *DIRS1*, *Gypsy*, and *Copia* families were highly conserved across the genera examined. To establish their phylogenetic relationships with respect to TEs from other eukaryotes, we first generated a majority rule nucleotidic consensus for each family of *DIRS1*, *Gypsy* and *Copia* TEs using Geneious v9.0.2 (http://www.geneious.com, [105]), then translated them to generate corresponding amino acid sequences. For *DIRS1* elements, the four nototheniid consensus sequences were added to a dataset of *DIRS1* [27] that span 96 amino acid sequences of the RT/RH region and represent numerous eukaryote taxa (including Fungi, Crustaceans, Nematodes). Two phylogenetic analyses of the nine *Gypsy* consensus sequences were performed, the first covering the RT/RH region (110 amino acids) and the second the INT region (99 amino acids). Nototheniid consensus sequences were added to the *Gypsy* sequence compilation [67]. Similarly, *CoNotoA* and *CoNotoB* sequences that span the RT/RH region were compared to a *Copia* dataset [67]. The final datasets contained 282 informative sites for *DIRS1*, 167 for *Gypsy* RT/RH, 153 for *Gypsy* INT and 220 for *Copia*.

Multisequence alignments were performed with MAFFT v7 and ambiguously aligned sites were removed using Gblocks [106]. Neighbor-joining (NJ) phylogenies were obtained using MEGA 5.2.2 [107]. The best-fit model was selected with Topali v2, implemented using PhyML [108] and the JTT model [109] with gamma distribution. Maximum Likelihood (ML) phylogenetic reconstructions were obtained using RAxML [110] and the evolution model PROTGAMMALG. Support for individual clusters was evaluated using non-parametric bootstrapping [111] and 1000 bootstrap replicates. Nodes under 60% were collapsed.

We selected outgroups for each reconstruction according to previously published retroelement relationships [17, 23, 30, 86]. Due to the proximity (sequence identity) of their RT/RH domains, we chose the *DIRS1* outgroup for the *Gypsy* reconstruction and reciprocally, *Gypsy* as outgroup for the *DIRS1* and *Copia* analyses. Because *DIRS1* elements do not encode an Integrase (but a tYrosine Recombinase), *Copia* (*GalEa*) sequences were used as root for the *Gypsy* analysis based on Integrase consensus fragments.

### FISH

#### TE probe preparation

*DIRS1*, *Gypsy* and *Copia* clones from *T. bernacchii*, *T. pennellii* and *N. coriiceps* greater than 1 kb in length were used as probes for FISH experiments (Additional file 6). TE probes were biotinylated by nick translation according to the manufacturer’s instructions (Roche Diagnostics). For the double FISH mapping, we used probes in which fluorochromes were directly incorporated during the labeling step; Fluorescein for *YNotoJ*, and Rhodamine for *YNotoR*, using the ULS Platinium*Bright* Nucleic Acid Labeling Kit (Leica Biosystems). Each probe was dissolved at a final concentration of 20 ng/μl in high stringency hybridization buffer [65% formamide, 2× SSC, 10% dextran sulfate (pH 7)].

#### FISH with TE probes

To ensure signal specificity for the three TE superfamilies, FISH was performed on chromosome preparations under high stringency conditions. Chromosome preparations were obtained from eight fish species, including five nototheniids (*T. pennelli*, *T. hansoni*, *T. eulepidotus*, *N. coriiceps*, *D. mawsoni*) and the three outgroup species (*B. diacanthus*, *E. maclovinus*, and *P. fluviatilis*). Biotinylated TE probes, denatured by heating at 85 °C for 5 min, were applied to freshly thawed chromosome preparations, which were then incubated at 72 °C for ten seconds to one minute. Bound probes were detected using Avidin-FITC (fluorescein) according to the protocol of Bonillo et al. [112], which is optimized for repetitive sequences and multi-copy genes. Hybridization parameters were adjusted to each chromosome preparation: we pre-incubated the slides at 37 °C to make chromosomes more resistant to denaturation, determined specific chromosome denaturation times for each specimen and tested several probe concentrations to select the one most adapted to our chromosomal material [113, 114].

#### Image acquisition and karyotyping

FISH signals were detected using a Zeiss Axioplan microscope equipped with a cooled CCD camera (Coolsnap Photometrics) and an XCite LED fluorescence light source. Karyotypes were processed using CytoVision 3.93.2/Genus FISH-imaging software for animal chromosomes (Leica Microsystems). Ten to forty metaphase spreads/species for each probe were examined.

### Quantification

#### Genome size determination

The flow cytometry procedure was based on Detrich et al. [60]. 500 μl of each sample was washed twice by centrifugation (Eppendorf 5430 microcentrifuge, 1500 rpm, 4 °C, 5 min), first in 0.01 M PBS followed by 0.01 M PBS containing RNAse (10 μg/ml, Miltenyi Biotec) and propidium iodide (PI; 50 μg/ml, Sigma Aldrich). Flow cytometry measurements were performed using a MACSQuant 10 flow cytometer. Unstained cells were used to determine autofluorescence thresholds and to calibrate the acquisition mode. Rainbow trout (*Oncorhynchus mykiss*) blood was collected at the Institut National de la Recherche Agronomique (INRA), Jouy-en-Josas (“synthetic INRA” strain, 2015). This rainbow trout provides our genome size reference, as it has been repeatedly estimated by flow cytometry with a mean calculated C-value of 2.695 pg (see also Volff et al. [13]: 2600 Mbp with 978 Mbp = 1 pg). We systematically started each run with triplicate measurements of the reference rainbow trout, followed by *N. coriiceps* and *T. bernachii* prior to data acquisition for the other nototheniid samples (Additional file 1). At least 10,000 cells were measured per sample and each experiment was replicated 3X. Fluorescence intensities, which were analyzed using FlowJo v10 software, reflect PI intercalation into DNA and are directly proportional to genome size. The genome sizes were calculated by comparing the mean of fluorescence intensity (MFI) of each sample to the MFI of our reference.

#### TE copy number determination by quantitative PCR (qPCR)

qPCR reactions were performed in a CFX96 Touch Real-time PCR Detection System (Bio-Rad). Specific primer pairs were designed with Oligo Analyzer v1.2, 1) to amplify 450 – 500 bp fragments overlapping the RT/RH conserved region of *DIRS1*, *Gypsy* and *Copia* TEs, and 2) to amplify fragments of similar size from the single copy *RAG1* and *Rhodopsin* genes (Additional file 12). The best practice recommendations of Bustin et al. [115] were followed for sample preparation. PCR amplifications were performed in a final volume of 20 μl containing 4 μl of template DNA, 10 μmol of each primer, 10 μl of Sso Advanced Universal SYBR Green Supermix (Bio-Rad), and 4 μl of DNAse free water. The PCR thermal cycling protocol began with polymerase activation and DNA denaturation at 98 °C for 4 min, followed by 40 cycles with denaturation for 15 s at 98 °C and annealing/extension for 1.5 min at 62 °C. After amplification, melting curve analyses were performed between 65 and 95 °C to determine amplification product specificity, with the temperature increasing of 0.1 °C/s. SYBR Green fluorescence was measured during the annealing/extension step. All samples were analyzed in triplicate with negative controls (different combinations: without DNA and primers, with DNA but without primers and with primers but without DNA).

For *DIRS1* elements, we created a primer pair to quantify simultaneously both the *YNotoR* and *YNotoB* families due to their high similarity in the RT/RH region (Additional file 12). Standard curves were generated for *RAG1* and *Rhodopsin* in each run. We performed qPCR for all fragments (*RAG1*, *Rhodopsin*, *YNotoJ*, *YNotoV*, *YNotoRB*, *GyNotoA*, *GyNotoE* and *CoNotoB*) for five species: *T. pennellii*, *T. hansoni*, *T. eulepidotus*, *N. coriiceps*, and *D. mawsoni*. PCR products were systematically checked by sequencing to verify primer specificity (Genewiz). Four to five tenfold dilution standards were prepared fresh each time, ranging from 1 to 1 × 10^− 4^ ng/μl. For both single copy gene standards, at least four dilutions in triplicate were obtained and plotted against Ct values. We estimated copy numbers of *DIRS1*, *Gypsy* and *Copia* family relative to both single copy (RAG1 on the one hand, and Rhodopsin on the other hand) calibration measurements using Bio-Rad CFX Manager v3.1, by comparison of their respective C(t) values, normalized to the DNA concentration initially present in the sample dilution. The slope of the standard curves was calculated and the amplification efficiency (E) was estimated as E = (10^− 1^/slope)^− 1^ for each amplicon and each dilution. We measured efficiencies for both standards (mean of 98.2 ± 2.6%), and for the whole TE families (mean of 97.8 ± 2.1%). *Tm* of each studied amplicon was constant between runs (Additional file 12).

## Additional files

Additional file 1: Repeatability of measurements for flow cytometry values determining genome sizes. Mean of C-values triplicate measurements (pg) per specimens and per species. (PDF 112 kb)

Additional file 2: Detection of identified TEs among *Trematomus*, *N. coriiceps* and *D. mawsoni* genomes. Distribution of *DIRS1*, *Gypsy* and *Copia* TEs identified in *Trematomus* and nototheniid sister species. (PDF 105 kb)

Additional file 3: NJ bootstrap consensus tree for *DIRS1* based on the RT/RH amino acid sequences. Complement of Fig. 1. We positioned our four *DIRS1* family consensus sequences (*YNotoJ*, *V*, *R*, *B*) in the context of a larger diverse dataset composed of well-described TE families from numerous eukaryote genomes. (PDF 223 kb)

Additional file 4: NJ bootstrap consensus tree for *Gypsy* based on the amino acid sequences of the RT/RH (a) and INT (b) regions. Complement of Fig. 2. We positioned our nine *Gypsy* TE family consensus sequences (*GyNotoA*, *B*, *D*, *E*, *F*, *H*, *I*, *J*, *RT*) in the context of a larger diverse dataset composed of well-described TE families from numerous eukaryote genomes. (PDF 309 kb)

Additional file 5: NJ bootstrap consensus tree for *Copia* based on the RT/RH amino acid sequences. Complement of Fig. 3. We positioned our two *Copia* TE family consensus sequences (*CoNotoA*, *B*) in the context of a larger diverse dataset composed of well-described TE families from numerous eukaryote genomes. (PDF 176 kb)

Additional file 6: Summary of TEs probes for *FISH*. sum up (name, superfamily, family, species it comes from, kb size) of cloned sequences used as probes for Fluorescent in situ hybridization. (PDF 86 kb)

Additional file 7: Mapping of TEs on the chromosomes of five nototheniid species by FISH. FISH mapping of a second family of *DIRS1* (*YNotoR*) and *Gypsy* (*GyNotoE*) identified and largely distributed in nototheniid genomes. (PDF 256 kb)

Additional file 8: Double mapping of two *DIRS1* family representatives on chromosomes of *T. hansoni*. Double FISH with two *DIRS1* family representatives (*YNotoJ*, directly labeled with fluorescein, greenish spots, and *YNotoR*, directly labled with Rhodamine, red spot). They are presented on a same metaphase spread. Firstly, with signals of each *DIRS1* family separated, and secondly superimposed. (PDF 278 kb)

Additional file 9: Taxonomic sampling for tissues, chromosomal suspensions and blood cells used in this study. For “Materials section”. Sum up of all specimen samples used for this study per Family and per *Genus. Species* (sample type, field reference, voucher reference). (PDF 26 kb)

Additional file 10: Degenerated PCR primers used to amplify retroelements in nototheniid genomes. For “Methods section”. Sum up of degenerated primers (primer sequence, motif overlapped, fragment size (pb)) used to amplify *DIRS1*, *Gypsy* and *Copia* retrotransposons in nototheniid genomes. Exploration and “TE walking”. (PDF 212 kb)

Additional file 11: Specific PCR primers used to amplify retroelements in nototheniid genomes (complement of Additional file 10). for “Methods section”. Complement of Additional file 10. Sum up of specific primers (primer sequence and fragment size (pb)) used to amplify *DIRS1*, *Gypsy* and *Copia* retrotransposons in nototheniid genomes. Specific amplifications. (PDF 176 kb)

Additional file 12: Specific qPCR primer pairs. For “Methods section”. Sum up of specific primers (primer sequence, fragment size (bp), region of amplification, Tm of amplification (°C)) used to amplify single copy genes *RAG1* and *Rhodopsin*, and *DIRS1*, *Gypsy* and *Copia* retrotransposons in nototheniid genomes for TE copy number quantification. (PDF 149 kb)

## Abbreviations

(q)PCR: (quantitative) Polymerase chain reaction
AFGP: Antifreeze glycoprotein
BAC: Bacterial artificial chromosome
DAPI: 4′,6-diamidino-2-phenylindole
DIG: Digoxigenin
FISH: Fluorescent in situ hybridization
FITC: Fluorescein isothiocyanate
ICR: Internal complementary region
INRA: Institut National de la Recherche Agronomique
ITR: Inversed terminal repeat
MFI: Mean on fluorescence intensity
MT: Methyltranferase
My: Million years
NJ: Neighbor joining
ORFs: Open reading frames
PBS: Phosphate buffered saline
RH: RNAseH
RT: Reverse transcriptase
SSC: Sodium chloride-sodium citrate buffer
TEs: Transposable elements
TSD: Target site duplication
YR: Tyrosine recombinase
