# Supplementary material for: Mobilization of retrotransposons as a cause of chromosomal diversification and rapid speciation: the case for the Antarctic teleost genus Trematomus
Source: BMC Genomics. 2018 May 9;19:339. doi: 10.1186/s12864-018-4714-x (PMC5941688; doi:10.1186/s12864-018-4714-x)
Supplement: Supplementary file 1 — Repeatability of measurements for flow cytometry values determining genome sizes. Mean of C-values triplicate measurements (pg) per specimens and per species. (PDF 112 kb) [file 12864_2018_4714_MOESM1_ESM.pdf]

Additional file 1: Repeatability of measurements for flow cytometry values determining genome sizes.

Sub-family	Genus. species	Field reference	C value (pg) mean / triplicate	Measure STDEVA	Mean STDEVA
Trematominae	*Teu*	CE 4011	1.261	0.013	
	Tpe	CE 4313	1.145	0.016	
	Tbo	CE 5560	1.052	0.024	0.017
		CE 5561	1.078	0.023	
		CE 5562	0.994	0.018	
		CE 5656	1.061	0.018	
		CE 5655	0.970	0.011	
		CE 5662	1.174	0.004	
		CE 5664	1.313	0.031	
		CE 5662	1.124	0.003	
	Tha	CE 4002	1.257	0.015	0.010
		CE 4003	1.271	0.016	
		CE 4004	1.331	0.012	
		CE 4316	1.193	0.008	
		CE 5708	1.154	0.009	
		CE 5711	1.052	0.016	
		CE 5738	1.248	0.007	
		CE 5856	1.320	0.005	
		CE 5880	1.307	0.005	
		CE 5660 (M)	1.288	0.008	
		CE 5661 (F)	1.304	0.008	
		CE 5664	1.364	0.010	
	Tbe	Ich 1098	1.059	0.005	0.014
		Ich 1099	1.231	0.016	
		Ich 1100	1.081	0.020	
		Ich 1101	1.257	0.010	
		Ich 1102	1.141	0.021	
		Ich 1114	1.213	0.000	
		CE 4315	1.087	0.031	
		CE 4314	1.178	0.007	
		CE 5709	1.096	0.016	
		CE 5737	1.051	0.026	
		CE 6042	1.093	0.045	
		CE 6043	1.076	0.003	
		CE 6091	1.118	0.002	
		CE 6092	1.033	0.005	
		CE 5658	1.100	0.014	
		CE 5669	1.238	0.010	
		CE 5685	0.991	0.004	
	Tlo	CE 5916	1.340	0.004	
	Tne	CE 4319	1.007	0.021	0.018
		TA 456	1.289	0.014	
	Tni	Ich 1115 (F)	1.206	0.007	0.008
		CE 5684	1.127	0.009	
Nototheniinae	*Nco*	Ich 1095 (F)	1.432	0.099	0.049
		Ich 1096 (F)	1.404	0.034	
		Ich 1097 (F)	1.236	0.013	
Dissostichinae	*Dma*	CE 4054 (M)	1.001	0.036	0.019
		CE 4055 (F)	1.004	0.009	
		CE 4056 (F)	1.050	0.011	
**Mean**			1.168	0.015	0.017

When identified, the sex of the specimen is indicated next to the field number M: male, F: female.
