# Supplementary material for: Mobilization of retrotransposons as a cause of chromosomal diversification and rapid speciation: the case for the Antarctic teleost genus Trematomus
Source: BMC Genomics. 2018 May 9;19:339. doi: 10.1186/s12864-018-4714-x (PMC5941688; doi:10.1186/s12864-018-4714-x)
Supplement: Supplementary file 2 — Detection of identified TEs among Trematomus, N. coriiceps and D. mawsoni genomes. Distribution of DIRS1, Gypsy and Copia TEs identified in Trematomus and nototheniid sister species. (PDF 105 kb) [file 12864_2018_4714_MOESM2_ESM.pdf]

Additional file 2: **Detection of identified TEs among *Trematomus*, *N. coriiceps* and *D. mawsoni* genomes**.

**Species**	***DIRS1 (YNoto)***				***Gyspy (GyNoto)***									***Copia (CoNoto)***	
	YB	*YJ*	*YR*	*YV*	*Gy A*	*Gy B*	*Gy D*	*Gy E*	*Gy F*	*Gy H*	*Gy I*	*Gy J*	*Gy RT*	*Co A*	*Co B*
T. eulepidotus	+	+	+	+	+	+	0	+	+	+	+	0	+	0	+
T. pennellii	+	+	+	+	+	+	0	+	+	+	+	0	+	0	+
T. lepidothinus	+	+	+	+	+	+	0	+	+	+	+	+	+	0	+
T. borchgrevinki	+	+	+	+	+	+	0	+	+	+	+	+	+	0	+
T. hansoni	+	+	+	+	+	+	0	+	+	+	+	0	+	0	+
T. bernacchii	+	+	+	+	+	+	0	+	+	+	+	0	+	0	+
T. loennbergi	+	+	+	+	+	+	0	+	+	+	+	0	+	0	+
T. newnesi	+	+	+	+	+	+	0	+	+	+	+	0	+	0	+
T. scotti	+	+	+	+	+	+	0	+	+	+	+	0	+	0	+
T. nicolaï	+	+	+	+	+	+	0	+	+	+	+	0	+	0	+
I. cyanobrancha	+	+	+	+	+	+	0	+	+	+	+	+	+	0	+
N. coriiceps	+	+	+	+	+	+	+	+	+	+	+	0	+	+	+
D. mawsoni	+	+	+	+	+	+	0	+	+	+	+	0	+	+	+

+ presence, 0 undetected
