# Supplementary material for: Mobilization of retrotransposons as a cause of chromosomal diversification and rapid speciation: the case for the Antarctic teleost genus Trematomus
Source: BMC Genomics. 2018 May 9;19:339. doi: 10.1186/s12864-018-4714-x (PMC5941688; doi:10.1186/s12864-018-4714-x)
Supplement: Supplementary file 3 — NJ bootstrap consensus tree for DIRS1 based on the RT/RH amino acid sequences. Complement of Fig. 1. We positioned our four DIRS1 family consensus sequences (YNotoJ, V, R, B) in the context of a larger diverse dataset composed of well-described TE families from numerous eukaryote genomes. (PDF 223 kb) [file 12864_2018_4714_MOESM3_ESM.pdf]

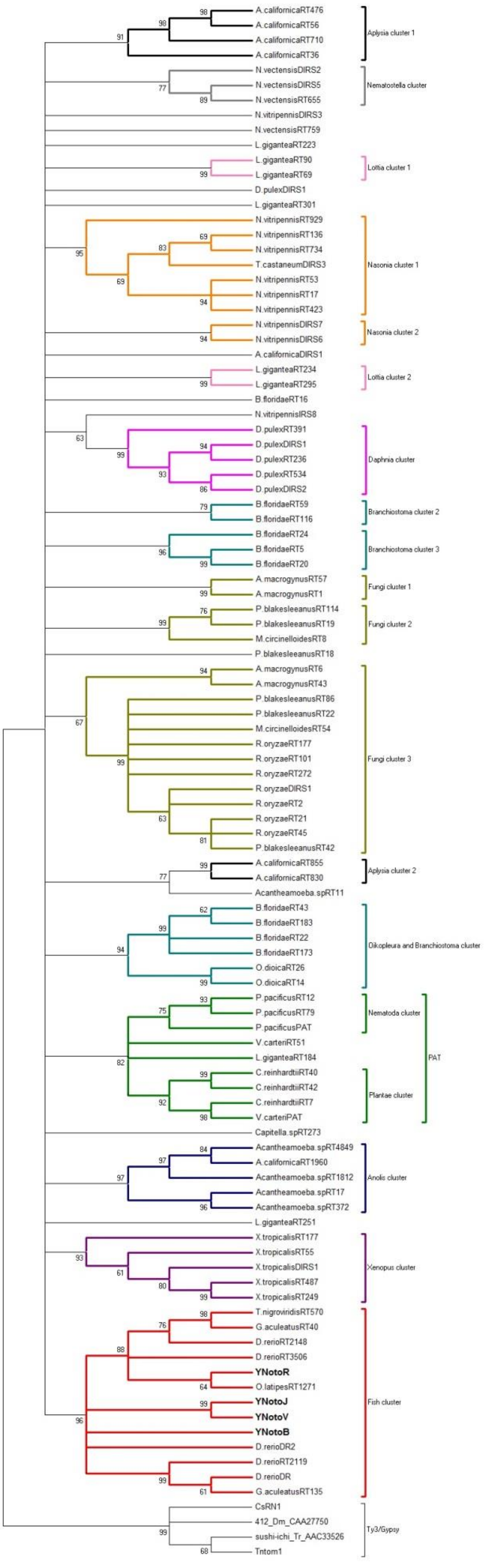

A.californicaRT476
A.californicaRT56
A.californicaRT710
A.californicaRT36
Aplysia cluster 1
N.vectensisDIRS2
N.vectensisDIRS5
N.vectensisRT655
Nematostella cluster
N.vitripennisDIRS3
N.vectensisRT759
L.giganteaRT223
L.giganteaRT90
L.giganteaRT69
Lottia cluster 1
D.pulexDIRS1
L.giganteaRT301
N.vitripennisRT929
N.vitripennisRT136
N.vitripennisRT734
T.castaneumDIRS3
N.vitripennisRT53
N.vitripennisRT17
N.vitripennisRT423
Nasonia cluster 1
N.vitripennisDIRS7
N.vitripennisDIRS6
Nasonia cluster 2
A.californicaDIRS1
L.giganteaRT234
L.giganteaRT295
Lottia cluster 2
B.floridaeRT16
N.vitripennisIRS8
D.pulexRT391
D.pulexDIRS1
D.pulexRT236
D.pulexRT534
D.pulexDIRS2
Daphnia cluster
B.floridaeRT59
B.floridaeRT116
Branchiostoma cluster 2
B.floridaeRT24
B.floridaeRT5
B.floridaeRT20
Branchiostoma cluster 3
A.macrogynusRT57
A.macrogynusRT1
Fungi cluster 1
P.blakesleeanusRT114
P.blakesleeanusRT19
M.circinelloidesRT8
Fungi cluster 2
P.blakesleeanusRT18
A.macrogynusRT6
A.macrogynusRT43
P.blakesleeanusRT86
P.blakesleeanusRT22
M.circinelloidesRT54
R.oryzaeRT177
R.oryzaeRT101
R.oryzaeRT272
R.oryzaeDIRS1
R.oryzaeRT2
R.oryzaeRT21
R.oryzaeRT45
P.blakesleeanusRT42
Fungi cluster 3
A.californicaRT855
A.californicaRT830
Aplysia cluster 2
Acantheamoeba.spRT11
B.floridaeRT43
B.floridaeRT183
B.floridaeRT22
B.floridaeRT173
O.dioicaRT26
O.dioicaRT14
Oikopleura and Branchiostoma cluster
P.pacificusRT12
P.pacificusRT79
P.pacificusPAT
Nematoda cluster
V.carteriRT51
L.giganteaRT184
C.reinhardtiiRT40
C.reinhardtiiRT42
C.reinhardtiiRT7
V.carteriPAT
Plantae cluster
PAT
Capitella.spRT273
Acantheamoeba.spRT4849
A.californicaRT1960
Acantheamoeba.spRT1812
Acantheamoeba.spRT17
Acantheamoeba.spRT372
Anolis cluster
L.giganteaRT251
X.tropicalisRT177
X.tropicalisRT55
X.tropicalisDIRS1
X.tropicalisRT487
X.tropicalisRT249
Xenopus cluster
T.nigroviridisRT570
G.aculeatusRT40
D.rerioRT2148
D.rerioRT3506
YNotoR
O.latipesRT1271
YNotoJ
YNotoV
YNotoB
D.rerioDR2
D.rerioRT2119
D.rerioDR
G.aculeatusRT135
Fish cluster
CsRN1
412_Dm_CAA27750
sushi-ichi_Tr_AAC33526
Tntom1
Ty3/Gypsy

Additional file 3: NJ bootstrap consensus tree for *DIRS1* based on the RT/RH amino acid sequences. Complement of Figure 1. The four families identified in nototheniid genomes (**bold font**) group with the other bony fish *DIRS1* sequences: *Tetraodon nigroviridis, Gasterosteus aculeatus* , *Danio rerio, Oryzias latipes*. Distances were calculated with the JTT model and a gamma distribution correction for amino acid. Support for individual clusters was evaluated using non-parametric bootstrapping with 1 000 replicates. Only bootstraps over 60 are presented. Nodes with bootstraps <60% were collapsed.
