# Supplementary material for: Mobilization of retrotransposons as a cause of chromosomal diversification and rapid speciation: the case for the Antarctic teleost genus Trematomus
Source: BMC Genomics. 2018 May 9;19:339. doi: 10.1186/s12864-018-4714-x (PMC5941688; doi:10.1186/s12864-018-4714-x)
Supplement: Supplementary file 4 — NJ bootstrap consensus tree for Gypsy based on the amino acid sequences of the RT/RH (a) and INT (b) regions. Complement of Fig. 2. We positioned our nine Gypsy TE family consensus sequences (GyNotoA, B, D, E, F, H, I, J, RT) in the context of a larger diverse dataset composed of well-described TE families from numerous eukaryote genomes. (PDF 309 kb) [file 12864_2018_4714_MOESM4_ESM.pdf]

a)

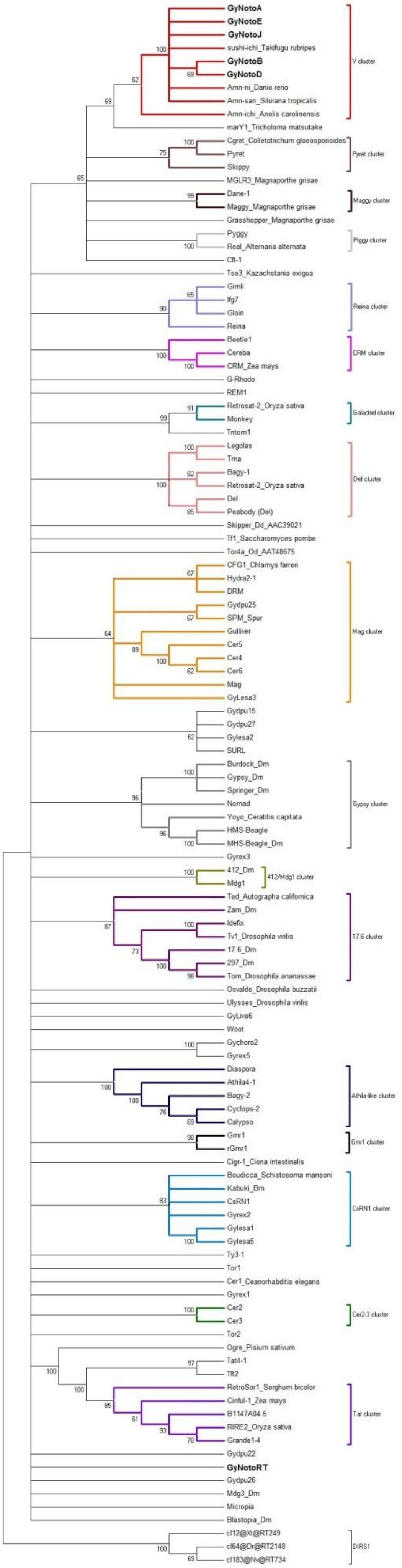

GyNotoA
GyNotoE
GyNotoJ
sushi-ichi_Takifugu rubripes
GyNotoB
GyNotoD
Amn-ni_Danio rerio
Amn-san_Silurana tropicalis
Amn-ichi_Anolis carolinensis
marY1_Tricholoma matsutake
V cluster
Cgret_Colletotrichum gloeosporioides
Pyret
Skippy
Pyret cluster
MGLR3_Magnaporthe grisae
Dane-1
Maggy_Magnaporthe grisae
Maggy cluster
Grasshopper_Magnaporthe grisae
Pyggy
Real_Alternaria alternata
Piggy cluster
Cft-1
Tse3_Kazachstania exigua
Gimli
Ifg7
Gloin
Reina
Reina cluster
Beetle1
Cereba
CRM_Zea mays
CRM cluster
G-Rhodo
REM1
Retrosat-2_Oryza sativa
Monkey
Galadriel cluster
Tntom1
Legolas
Tma
Bagy-1
Retrosat-2_Oryza sativa
Del
Peabody (Del)
Del cluster
Skipper_Dd_AAC39021
Tf1_Saccharomyces pombe
Tor4a_Od_AAT48675
CFG1_Chlamys farreri
Hydra2-1
DRM
Gydpu25
SPM_Spur
Gulliver
Cer5
Cer4
Cer6
Mag
GyLesa3
Mag cluster
Gydpu15
Gydpu27
Gylesa2
SURL
Burdock_Dm
Gypsy_Dm
Springer_Dm
Nomad
Yoyo_Ceratitis capitata
HMS-Beagle
MHS-Beagle_Dm
Gypsy cluster
Gyrex3
412_Dm
Mdg1
412/Mdg1 cluster
Ted_Autographa californica
Zam_Dm
Idefix
Tv1_Drosophila virilis
17.6_Dm
297_Dm
Tom_Drosophila ananassae
17.6 cluster
Osvaldo_Drosophila buzzatii
Ulysses_Drosophila virilis
GyLiva6
Woot
Gychoro2
Gyrex5
Diaspora
Athila4-1
Bagy-2
Cyclops-2
Calypso
Athila-like cluster
Gmr1
rGmr1
Gmr1 cluster
Cigr-1_Ciona intestinalis
Boudicca_Schistosoma mansoni
Kabuki_Bm
CsRN1
Gyrex2
Gylesa1
Gylesa5
CsRN1 cluster
Ty3-1
Tor1
Cer1_Ceanorhabditis elegans
Gyrex1
Cer2
Cer3
Cer2-3 cluster
Tor2
Ogre_Pisium sativum
Tat4-1
Tft2
RetroSor1_Sorghum bicolor
Cinful-1_Zea mays
B1147A04.5
RIRE2_Oryza sativa
Grande1-4
Tat cluster
Gydpu22
GyNotoRT
Gydpu26
Mdg3_Dm
Micropia
Blastopia_Dm
cl12@Xt@RT249
cl64@Dr@RT2148
cl183@Nv@RT734
DIRS1

b)

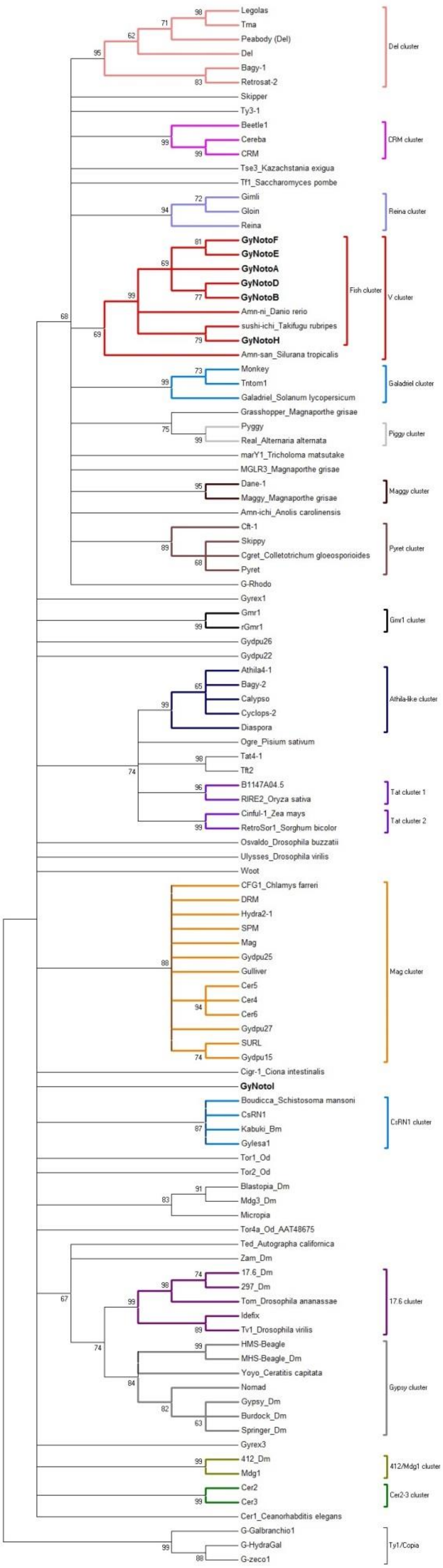

Legolas
Tma
Peabody (Del)
Del
Bagy-1
Retrosat-2
Skipper
Ty3-1
Beetle1
Cereba
CRM
Tse3_Kazachstania exigua
Tf1_Saccharomyces pombe
Gimli
Gloin
Reina
GyNotoF
GyNotoE
GyNotoA
GyNotoD
GyNotoB
Amn-ni_Danio rerio
sushi-ichi_Takifugu rubripes
GyNotoH
Amn-san_Silurana tropicalis
Monkey
Tntom1
Galadriel_Solanum lycopersicum
Grasshopper_Magnaporthe grisae
Pyggy
Real_Alternaria alternata
marY1_Tricholoma matsutake
MGLR3_Magnaporthe grisae
Dane-1
Maggy_Magnaporthe grisae
Amn-ichi_Anolis carolinensis
Cft-1
Skippy
Cgret_Colletotrichum gloeosporioides
Pyret
G-Rhodo
Gyrex1
Gmr1
rGmr1
Gydpu26
Gydpu22
Athila4-1
Bagy-2
Calypso
Cyclops-2
Diaspora
Ogre_Pisium sativum
Tat4-1
Tft2
B1147A04.5
RIRE2_Oryza sativa
Cinful-1_Zea mays
RetroSor1_Sorghum bicolor
Osvaldo_Drosophila buzzatii
Ulysses_Drosophila virilis
Woot
CFG1_Chlamys farreri
DRM
Hydra2-1
SPM
Mag
Gydpu25
Gulliver
Cer5
Cer4
Cer6
Gydpu27
SURL
Gydpu15
Cigr-1_Ciona intestinalis
GyNotoI
Boudicca_Schistosoma mansoni
CsRN1
Kabuki_Bm
Gylesa1
Tor1_Od
Tor2_Od
Blastopia_Dm
Mdg3_Dm
Micropia
Tor4a_Od_AAT48675
Ted_Autographa californica
Zam_Dm
17.6_Dm
297_Dm
Tom_Drosophila ananassae
Idefix
Tv1_Drosophila virilis
HMS-Beagle
MHS-Beagle_Dm
Yoyo_Ceratitis capitata
Nomad
Gypsy_Dm
Burdock_Dm
Springer_Dm
Gyrex3
412_Dm
Mdg1
Cer2
Cer3
Cer1_Ceanorhabditis elegans
G-Galbranchio1
G-HydraGal
G-zeco1
Del cluster
CRM cluster
Reina cluster
Fish cluster
V cluster
Galadriel cluster
Piggy cluster
Maggy cluster
Pyret cluster
Gmr1 cluster
Athila-like cluster
Tat cluster 1
Tat cluster 2
Mag cluster
CsRN1 cluster
17.6 cluster
Gypsy cluster
412/Mdg1 cluster
Cer2-3 cluster
Ty1/Copia

Additional file 4: NJ bootstrap consensus tree for Gypsy based on the amino acid sequences of the RT/RH (a) and INT (b) regions. Complement of Figure 2. Except for *GyNotoI* and *GyNotoRT*, the families identified in nototheniid genomes (**bold font**) group with the other vertebrate: *Xenopus*/*Silurana tropicalis*; and particularly with bony fish *Gypsy* sequences: *Tetraodon rubripes* -sushi-ichi, *Danio rerio* -Amn-ni. Distances were calculated with the JTT model and the gamma distribution correction for amino acid. Support for individual clusters was evaluated using non-parametric bootstrapping with 1 000 replicates. Only bootstraps over 60 are presented. Nodes with bootstraps <60% were collapsed.
