# Supplementary material for: Mobilization of retrotransposons as a cause of chromosomal diversification and rapid speciation: the case for the Antarctic teleost genus Trematomus
Source: BMC Genomics. 2018 May 9;19:339. doi: 10.1186/s12864-018-4714-x (PMC5941688; doi:10.1186/s12864-018-4714-x)
Supplement: Supplementary file 5 — NJ bootstrap consensus tree for Copia based on the RT/RH amino acid sequences. Complement of Fig. 3. We positioned our two Copia TE family consensus sequences (CoNotoA, B) in the context of a larger diverse dataset composed of well-described TE families from numerous eukaryote genomes. (PDF 176 kb) [file 12864_2018_4714_MOESM5_ESM.pdf]

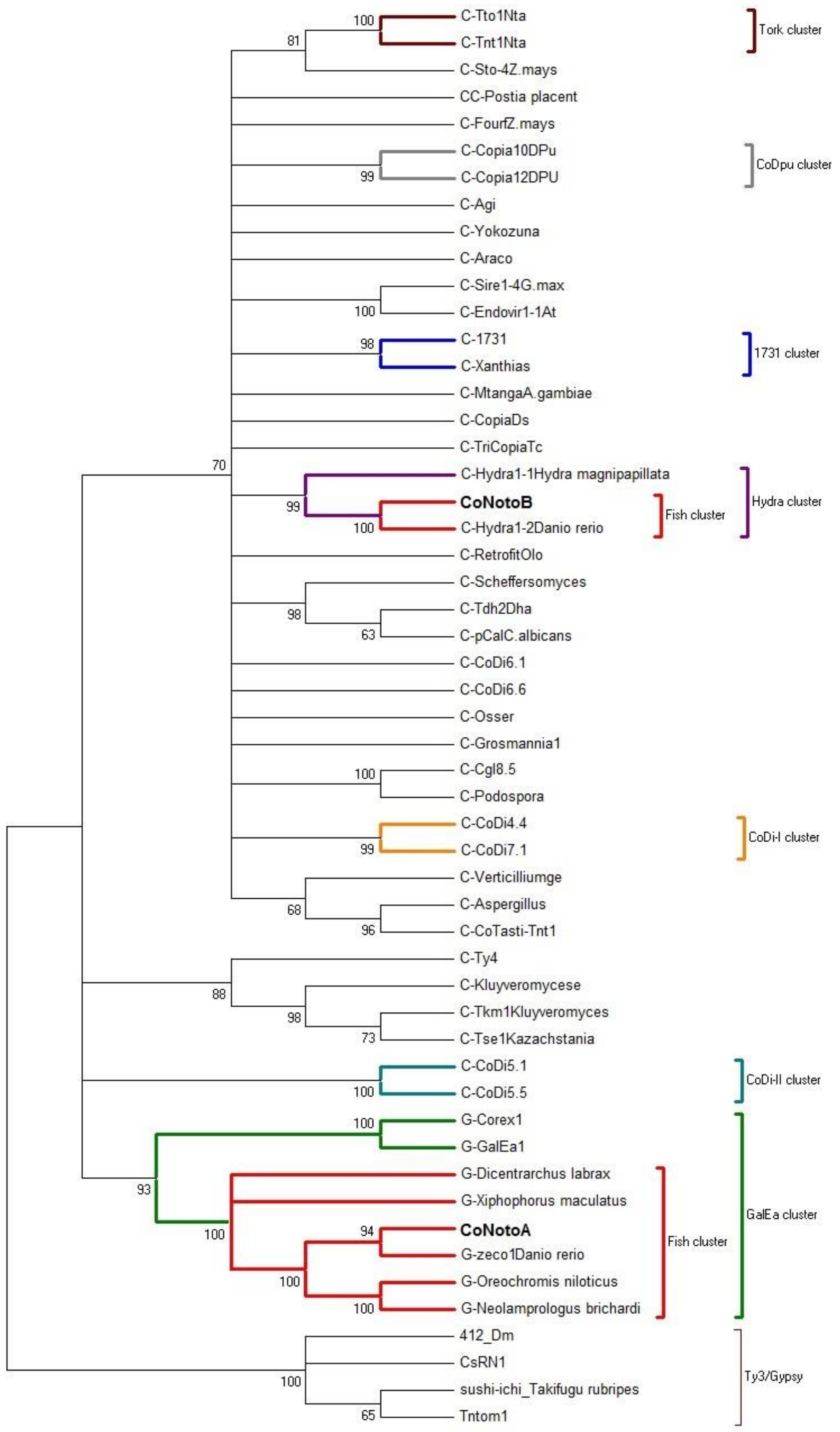
C-Tto1Nta
C-Tnt1Nta
C-Sto-4Z.mays
CC-Postia placent
C-FourfZ.mays
C-Copia10DPu
C-Copia12DPU
C-Agi
C-Yokozuna
C-Araco
C-Sire1-4G.max
C-Endovir1-1At
C-1731
C-Xanthias
C-MtangaA.gambiae
C-CopiaDs
C-TriCopiaTc
C-Hydra1-1Hydra magnipapillata
CoNotoB
C-Hydra1-2Danio rerio
C-RetrofitOlo
C-Scheffersomyces
C-Tdh2Dha
C-pCalC.albicans
C-CoDi6.1
C-CoDi6.6
C-Osser
C-Grosmannia1
C-Cgl8.5
C-Podospora
C-CoDi4.4
C-CoDi7.1
C-Verticilliumge
C-Aspergillus
C-CoTasti-Tnt1
C-Ty4
C-Kluyveromycese
C-Tkm1Kluyveromyces
C-Tse1Kazachstania
C-CoDi5.1
C-CoDi5.5
G-Corex1
G-GalEa1
G-Dicentrarchus labrax
G-Xiphophorus maculatus
CoNotoA
G-zeco1Danio rerio
G-Oreochromis niloticus
G-Neolamprologus brichardi
412_Dm
CsRN1
sushi-ichi_Takifugu rubripes
Tntom1
Tork cluster
CoDpu cluster
1731 cluster
Hydra cluster
Fish cluster
CoDi-I cluster
CoDi-II cluster
GalEa cluster
Fish cluster
Ty3/Gypsy
100
81
99
100
98
70
99
100
98
63
100
99
68
96
88
98
73
100
100
93
100
94
100
100
100
65

Additional file 5: NJ bootstrap consensus tree for *Copia* based on the RT/RH amino acid sequences. Complement of Figure 3. The *GalEa* and *Hydra* families identified in nototheniid genomes (**bold font**) group with the other bony fish *Copia* sequences: *Dicentrarchus labrax*, *Xiphophorus maculatus*, *Danio rerio*, *Oreochromis niloticus* and *Neolamprologus brichardi*. Distances were calculated with the JTT model and the gamma distribution correction for amino acid. Support for individual clusters was evaluated using non-parametric bootstrapping with 1 000 replicates. Only bootstraps over 60 are presented. Nodes with bootstraps <60% were collapsed.
