# Supplementary material for: Mobilization of retrotransposons as a cause of chromosomal diversification and rapid speciation: the case for the Antarctic teleost genus Trematomus
Source: BMC Genomics. 2018 May 9;19:339. doi: 10.1186/s12864-018-4714-x (PMC5941688; doi:10.1186/s12864-018-4714-x)
Supplement: Supplementary file 6 — Summary of TEs probes for FISH. sum up (name, superfamily, family, species it comes from, kb size) of cloned sequences used as probes for Fluorescent in situ hybridization. (PDF 86 kb) [file 12864_2018_4714_MOESM6_ESM.pdf]

Additional file 6: **Summary of TEs probes for *FISH***

TEs	Family	Sequence name	Species related	N° specimen*	Insert size (kb)
DIRS1	*YNotoJ*	*YTbeJ1*	*T. bernacchii*	TA364TRPE1	1.25
	YNotoR	*YThaR1*	*T. hansoni*	TA440TRHA2	1.10
Gypsy	*GyNotoA*	*GyTpeA1*	*T. pennellii*	TA364TRPE1	1.54
	GyNotoE	*GyThaE1*	*T. hansoni*	TA440TRHA2	1.50
Copia	*CoNotoB (Hydra)*	*CoTpeB1*	*T. pennellii*	TA364TRPE1	1.10

* for precisions (origin, survey), see Additional file 9 (taxonomic sampling). For precision about TE sequence characteristics, see Table 2.
