# Supplementary material for: Mobilization of retrotransposons as a cause of chromosomal diversification and rapid speciation: the case for the Antarctic teleost genus Trematomus
Source: BMC Genomics. 2018 May 9;19:339. doi: 10.1186/s12864-018-4714-x (PMC5941688; doi:10.1186/s12864-018-4714-x)
Supplement: Supplementary file 7 — Mapping of TEs on the chromosomes of five nototheniid species by FISH. FISH mapping of a second family of DIRS1 (YNotoR) and Gypsy (GyNotoE) identified and largely distributed in nototheniid genomes. (PDF 256 kb) [file 12864_2018_4714_MOESM7_ESM.pdf]

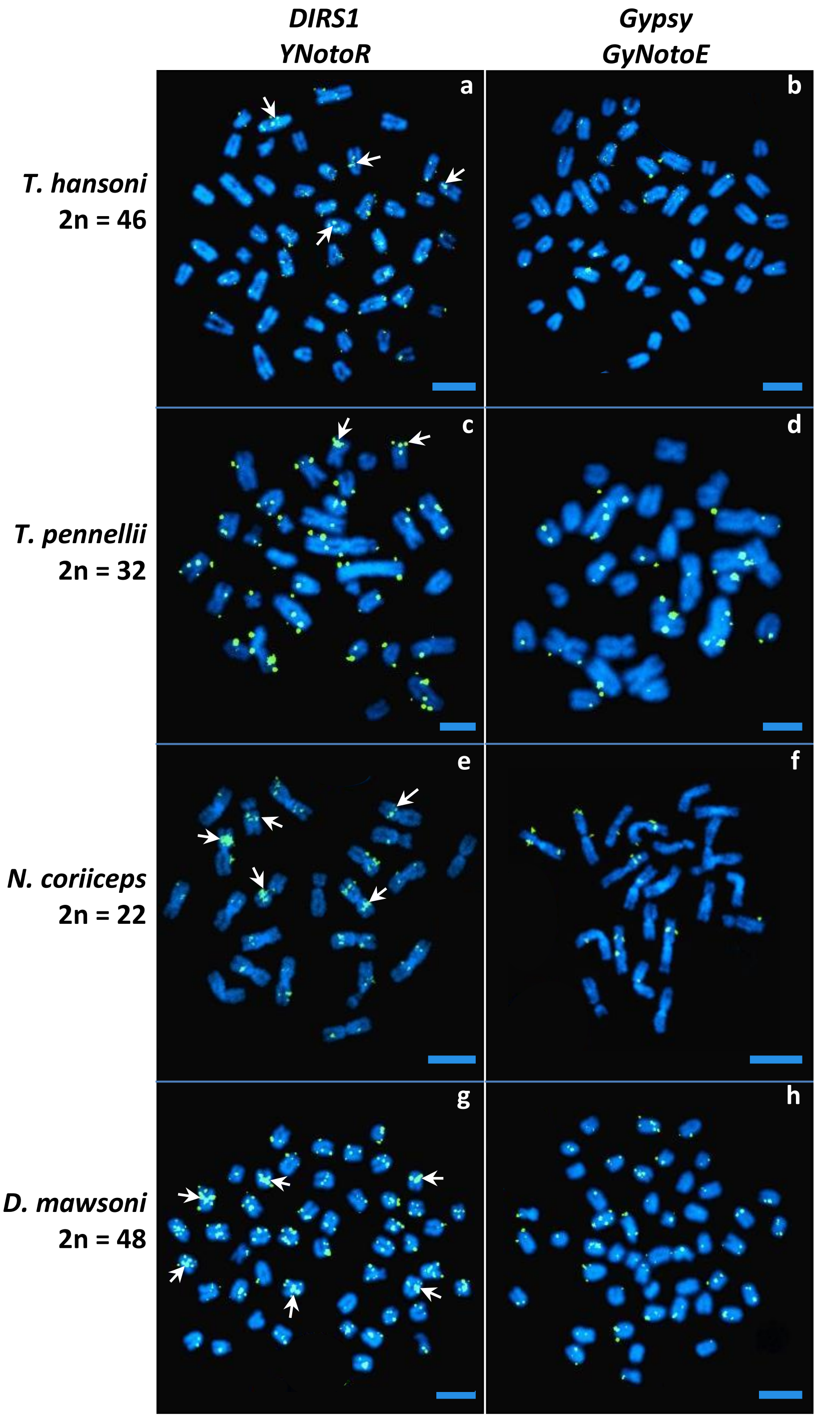

Additional file 7: Mapping of TEs on the chromosomes of five nototheniid species by FISH. Complement of Figure 4. Each probe was labeled with biotin and bound probe was detected with incubation with Avidin-FITC (fluorescein, greenish spots). (Probe characteristics are indicated in Additional file 6). Chromosomal DNA was counterstained with DAPI. One family of each retrotransposon superfamily is represented in this figure: *YNotoR* for *DIRS1*, *GyNotoE* for *Gypsy* elements. White arrows point examples of TE accumulations (distribution pattern corresponding to type 1). Scale bars: 10 µm.
