# Supplementary material for: Mobilization of retrotransposons as a cause of chromosomal diversification and rapid speciation: the case for the Antarctic teleost genus Trematomus
Source: BMC Genomics. 2018 May 9;19:339. doi: 10.1186/s12864-018-4714-x (PMC5941688; doi:10.1186/s12864-018-4714-x)
Supplement: Supplementary file 8 — Double mapping of two DIRS1 family representatives on chromosomes of T. hansoni. Double FISH with two DIRS1 family representatives (YNotoJ, directly labeled with fluorescein, greenish spots, and YNotoR, directly labled with Rhodamine, red spot). They are presented on a same metaphase spread. Firstly, with signals of each DIRS1 family separated, and secondly superimposed. (PDF 278 kb) [file 12864_2018_4714_MOESM8_ESM.pdf]

Additional file 8: Double mapping of two *DIRS1* family representatives on chromosomes of *T. hansoni*.

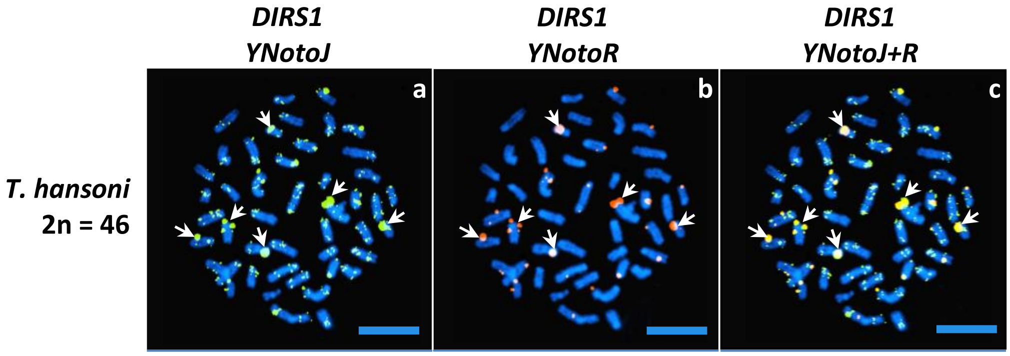

Complement of Figure 4 and Additional file 7. *YNotoJ* probe was directly labeled with fluorescein (greenish spots) and *YNotoR* probe, with Rhodamine (red spots). (Probe characteristics are indicated in Additional file 6). Chromosomal DNA was counterstained with DAPI. Two families of *DIRS1* elements are represented in this figure: *YNotoJ* (a), *YNotoR* (b), and the resulting signals of both of them superimposed (c). White arrows point examples of TE accumulations (distribution pattern corresponding to type 1), and the colocation of two *DIRS1* families. Scale bars: 10 µm.
