# Supplementary material for: Mobilization of retrotransposons as a cause of chromosomal diversification and rapid speciation: the case for the Antarctic teleost genus Trematomus
Source: BMC Genomics. 2018 May 9;19:339. doi: 10.1186/s12864-018-4714-x (PMC5941688; doi:10.1186/s12864-018-4714-x)
Supplement: Supplementary file 9 — Taxonomic sampling for tissues, chromosomal suspensions and blood cells used in this study. For “Materials section”. Sum up of all specimen samples used for this study per Family and per Genus. Species (sample type, field reference, voucher reference). (PDF 26 kb) [file 12864_2018_4714_MOESM9_ESM.pdf]

Additional file 9: **Taxonomic sampling for tissues, chromosomal suspensions and blood cells used in this study**

Family (sub-family)	*Genus. Species*	Sampling type	Field reference	Voucher reference
Nototheniidae (Trematominae)	*T. eulepidotus*	tissue	si487n3152[4]	MNHN 2009-1358
		chromosomes	CE2381[5], m251[6]	
		erythrocytes	CE4011 [5]	
	T. pennellii	tissue	TA364TRPE1[3]	MNHN 1996-0319
		chromosomes	TA647[3]	
		erythrocytes	CE1434 [5]	
	T. borchgrevinki	tissue	TA263PABO1[3]	MNHN 2002-1711
		erythrocytes	CE5560 / 5561 / 5562 / 5655 / 5656 / 5662 / 5664 [5]	
	T. hansoni	tissue	TA440TRHA2[3]	MNHN 1996-0307
		chromosomes	TA 646TRHA2[3]	
		erythrocytes	CE 4002 / 4003 / 4004 / 4316 / 5660 /5664 / 5708 / 5711 / 5738 / 5856 / 5880 / 5661) [5]	
	T. bernacchii	tissue	Si352n2561[4] / TA 337TRBE7-1/01/03[3]	MNHN 2009-1312
		erythrocytes	Ich1098 / 1099 / 1100 / 1101 / 1102 / 1114 / CE4314 / 4315 / 5658 / 5659 / 5685 / 5709 / 5737 / 6042 / 6043 / 6091 / 6092[5]	
	T. loennbergii	tissue	si259n923[4] / si170n1265[4]	MNHN 2009-1246
		erythrocytes	CE5916[5]	
	T. lepidorhinus	tissue	TNB238[6]	MNHN 1999-0394
	T. newnesi	tissue	TA401TRNE3[3]	MNHN 2001-1148[3,5]
		erythrocytes	CE4319[5] / TA456[3]	
	T. scotti	tissue	si541n6317[4]	MNHN 2009-1368
	T. nicolaï	tissue	TNB214[6]	MNHN 1999-0400[6]
		erythrocytes	Ich1115 / CE5684[5]	MNHN 2002-1707[5]
	I. cyanobrancha	tissue	Aus30[2]	MNHN 2007-1844
Nototheniidae (Nototheniinae)	*N. coriiceps*	tissue	N°10RECOLTE2001 / TA421NOCO8[3]	MNHN 2008-1884[3,4,5] not vouchered[1]
		chromosomes	TA449[3] / NC4[1]	
		erythrocytes	Ich1095 / 1096 / 1097[5]	
Nototheniidae (Dissostichinae)	*D. mawsoni*	tissue	REVO759[5]	MNHN 2001-1143[5] not vouchered[1]
		chromosomes	DM1[1]	
		erythrocytes	CE4054 / 4055 / 4056[5]	
Eleginopsidae	*E. maclovinus*	chromosomes	RG[1]	MNHN 2005-0093
Bovichtidae	*B. diacanthus*	chromosomes	BD1 / BD4[1]	MNHN 2005-0102
Percidae	*P. fluviatilis*	chromosomes		MNHN 2010-987

Tissue samples, chromosomal preparations and blood cells were collected during the Antarctic campaigns of the IPEV programs: **ICEFISH2**[1] (2004, Southern Ocean and south atlantic), **POKER**[2] (2010 and 2013 Kerguelen-Heard, shelf and islands), **ICOTA**[3] (1996-2008, Adelie Land), **CEAMARC**[4] (2007/2008 north of Adélie Land and George V Land of Eastern Antarctica), **REVOLTA**[5] (2010-2014, Adelie Land). The fish identifier and collector is Catherine Ozouf-Costaz except for NC4, DM1, RG, BD1 and BD4: Guillaume Lecointre;TNB and m251: Eva Pisano, and sample of *P. fluviatilis*: Gaël Denys. TNB and m251 samples are originated from the **ICEFISH1**[6] campaign and conserved in the museum of Genoa (no voucher reference). A single voucher reference is provided per species/locality. Other specimens are deposited in the MNHN research collections.
