# Supplementary material for: Mobilization of retrotransposons as a cause of chromosomal diversification and rapid speciation: the case for the Antarctic teleost genus Trematomus
Source: BMC Genomics. 2018 May 9;19:339. doi: 10.1186/s12864-018-4714-x (PMC5941688; doi:10.1186/s12864-018-4714-x)
Supplement: Supplementary file 10 — Degenerated PCR primers used to amplify retroelements in nototheniid genomes. For “Methods section”. Sum up of degenerated primers (primer sequence, motif overlapped, fragment size (pb)) used to amplify DIRS1, Gypsy and Copia retrotransposons in nototheniid genomes. Exploration and “TE walking”. (PDF 212 kb) [file 12864_2018_4714_MOESM10_ESM.pdf]

Additional file 10: **Degenerated PCR primers used to amplify retroelements in nototheniid genomes**

	TE	Primer	Primer sequence 5’ → 3’	Motif	Fragment size (kb)
**deg**	*Gypsy*	GD1b	KYYTBCCRTTYGGSYT	PFGL	0.7
		GD2b	CCCCAGCCYKWSYKHCARGC	DAS..GW	
	Copia Hydra	CD1	ARRGCNMGNYTNGTNGC	KARLVA	0.6
		CD3	AYRTNGAYTAYTGYTAYMG	DYCYR	1.4
		CD4	GCDATNGYNCCYTGRTTRTC	DNQG	
	Copia GalEa	CD5	GAYCCNGCNGTNTTYTAYTGG	VDP	0.9
		CD6	TNARRCARTCNGCNARYTG	QLAD	
	DIRS1	DD10	GAYYTNAARGAYGCNTAY	DlkdAY	1.2
		DD11	RAANGGNGGRAANRYRTA	YafPPf	
**spe-deg**	*Gypsy*	GD4	NYVDTAYMTVGAYGAY	YLDD	1.3

deg: degenerate primer pairs used to amplify the different *DIRS1*, *Gypsy* and *Copia* TE superfamilies.

spe-deg: degenerate primer coupled with specific primers (listed in Additional file 11) for the “TE walking”.
