# Supplementary material for: Mobilization of retrotransposons as a cause of chromosomal diversification and rapid speciation: the case for the Antarctic teleost genus Trematomus
Source: BMC Genomics. 2018 May 9;19:339. doi: 10.1186/s12864-018-4714-x (PMC5941688; doi:10.1186/s12864-018-4714-x)
Supplement: Supplementary file 11 — Specific PCR primers used to amplify retroelements in nototheniid genomes (complement of Additional file 10). for “Methods section”. Complement of Additional file 10. Sum up of specific primers (primer sequence and fragment size (pb)) used to amplify DIRS1, Gypsy and Copia retrotransposons in nototheniid genomes. Specific amplifications. (PDF 176 kb) [file 12864_2018_4714_MOESM11_ESM.pdf]

Additional file 11: **Specific PCR primers used to amplify retroelements in nototheniid genomes (complement of Additional file 9).**

	TE	Primer	Primer sequence 5’ → 3’	Fragment size (kb)
**spe***	*Gypsy*	GS1	ATGGGTAGAGGCTGAAGCAA	1.3
		GS2	GGAACTTTCCCTGGTGT	
		GS3	TCCACGGAAATGTATGACCA	
**spe**	*Gypsy*	GS4	GACGGAGAAGTGCGAGTTTC	1.6
		GS5	ATTGGCACAATGGAAAGGAG	
		GS6	GACGGAGAAGTGCGAGTTTC	1.5
		GS7	ATTGGCACAATGGAAAGGAG	
		GSA1	GGGTCAAGGAAGTGTGGAGA	1.5
		GSA2	CTCCACCCAMAGGAGTTGTT	
		GSB1	CCAGGAAGAAGGTYCARCAG	1.5
		GSB2	RCTCCAGGTRKTCTGGTTCYG	
		GSE1	CACGTTTCCACCGTCTTT	1.5
		GSEF2	CTGAAATGGGGAAAGACCAG	
		GSF1	AGGTGGGGGAGTATGTTGCT	0.6
		GSH1	TGTCAACGAGGACACTCAGG	0.6
		GSH2	CAAACGGGGAGAGACCAGTA	
		GSI1	TGCTTAACCCACAACATCAGA	0.5
		GSI2	AAATGGGGAGAAACCTGTCC	
		GSJ1	AGGCTGAAGCAAGCAGGTTA	1.5
		GSJ2	TAGAGGGGTTACGGTCAACG	
		GSRT1	CTAAGGGATGAGTGCTGCTC	0.6
		GSRT2	GAAGGACGAAGGGCAAA	
	Copia	HS1	GCCCAGATATGGTTGCAAAAGG	1.1
	Hydra	HS2	TGACCCTCACTRTCCATGCTCT	
	DIRS1	DSB1	CTCTCCCTCTCTCCAAGGGT	1.0
		DSB2	CATYGGGCTGGTCACCTCTG	
		DSR1	CATCCCARTATATTATCCAC	1.1
		DSR2	CGCATTGAAAAGAACAGCTG	
		DSJ1	TTCAGCAAATGTGTGGCCA	1.1
		DSJ2	CGTGTGCAAACGCGTCCAC	
		DSV1	CTGGCTCCTCGCACTTTTTC	1.0
		DSV2	GCGCGTTCTCGCGTGCAGC	

spe*: specific primers coupled with spe-deg primer (Additional file 10) for the “TE walking”.

spe: specific primer pairs used to amplify the different families of *DIRS1*, *Gypsy* and *Copia* TEs.
