# Supplementary material for: Mobilization of retrotransposons as a cause of chromosomal diversification and rapid speciation: the case for the Antarctic teleost genus Trematomus
Source: BMC Genomics. 2018 May 9;19:339. doi: 10.1186/s12864-018-4714-x (PMC5941688; doi:10.1186/s12864-018-4714-x)
Supplement: Supplementary file 12 — Specific qPCR primer pairs. For “Methods section”. Sum up of specific primers (primer sequence, fragment size (bp), region of amplification, Tm of amplification (°C)) used to amplify single copy genes RAG1 and Rhodopsin, and DIRS1, Gypsy and Copia retrotransposons in nototheniid genomes for TE copy number quantification. (PDF 149 kb) [file 12864_2018_4714_MOESM12_ESM.pdf]

## Additional file 12: Specific qPCR primer pairs

| Sequence type | Markers | primer | Primer sequence 5' → 3' | Length (bp) | Region of amplicon | Tm of amplicon (°C) |
|---|---|---|---|---|---|---|
| Single copy genes | *RAG1* | RAG1F23 | AGCCAGCTCTCAAGAATGTGTC | 457 | RAG1 | 86.0 |
| | | RAG1R458 | GTGAGTGTCTCATGGTCTGACT | | | |
| | *Rhodopsin* | RhodoF193 | CCNTATGAATAYCCTCAGTACTACC | 396 | Rhodo_N 7TM-GPCR | 86.5 |
| | | RhodoR563 | GTGATCCTCCCCGAAGCG | | | |
| DIRS1 | *YNotoJ* | DJF226 | CGGGAACAGGCAATGTTTTG | 475 | RT/RH | 89.0 |
| | | DJR676 | TCCGTGAACACTGTGATGT | | | |
| | *YNotoV* | DVF229 | GAAGTNGCTTTACAGACGGTACA | 447 | RT/RH | 88.5 |
| | | DVR656 | GTGACGTAACTCTGCCCAGG | | | |
| | *YNotoR/B* | DRBF185 | GCCGTATCTAGACRACTGGC | 530 | RT/RH | 89.3 |
| | | DRBR696 | CCCACCCASAGAGACATGC | | | |
| *Gypsy* | *GyNotoA* | GF1all | GACGGAGAAGTGCGAGTTTC | 503 | RT/RH | 88.0 |
| | | GR480g1 | CCAACGCCAACTTGACTGCT | | | |
| | *GyNotoE* | GyNotoEF330 | TTGACGCTTCWGATGTGGGCG | 452 | RT/RH | 86.5 |
| | | GyNotoER785 | RACCCTCTCCTCAATGTCCCA | | | |
| *Copia* | *CoNotoB* | HydraF951 | ACAATATCGTGAAGTGGTAGGCA | 431 | RT/RH | 83.5 |
| | | HydraR1391 | TGACCCTCACTRTCCATGCTCT | | | |
